# On the Species Delimitation of the *Maddenia* Group of *Prunus* (Rosaceae): Evidence From Plastome and Nuclear Sequences and Morphology

**DOI:** 10.3389/fpls.2021.743643

**Published:** 2021-10-11

**Authors:** Na Su, Bin-bin Liu, Jun-ru Wang, Ru-chang Tong, Chen Ren, Zhao-yang Chang, Liang Zhao, Daniel Potter, Jun Wen

**Affiliations:** ^1^College of Life Sciences, Northwest A&F University, Yangling, China; ^2^Herbarium of Northwest A&F University, Yangling, China; ^3^State Key Laboratory of Systematic and Evolutionary Botany, Institute of Botany, Chinese Academy of Sciences, Beijing, China; ^4^Department of Botany, National Museum of Natural History, MRC 166, Smithsonian Institution, Washington, DC, United States; ^5^Key Laboratory of Plant Resources Conservation and Sustainable Utilization, South China Botanical Garden, Chinese Academy of Sciences, Guangzhou, China; ^6^Center of Conservation Biology, Core Botanical Gardens, South China Botanical Garden, Chinese Academy of Sciences, Guangzhou, China; ^7^Department of Plant Sciences, MS2, University of California, Davis, Davis, CA, United States

**Keywords:** *Maddenia*, *Prunus*, Rosaceae, barcoding, chloroplast genome, single-copy nuclear genes, species delimitation

## Abstract

The recognition, identification, and differentiation of closely related plant species present significant and notorious challenges to taxonomists. The *Maddenia* group of *Prunus*, which comprises four to seven species, is an example of a group in which species delimitation and phylogenetic reconstruction have been difficult, due to the lack of clear morphological distinctions, limited sampling, and low informativeness of molecular evidence. Thus, the precise number of species in the group and the relationships among them remain unclear. Here, we used genome skimming to generate the DNA sequence data for 22 samples, including 17 *Maddenia* individuals and five outgroups in Amygdaloideae of Rosaceae, from which we assembled the plastome and 446 single-copy nuclear (SCN) genes for each sample. The phylogenetic relationships of the *Maddenia* group were then reconstructed using both concatenated and coalescent-based methods. We also identified eight highly variable regions and detected simple sequence repeats (SSRs) and repeat sequences in the *Maddenia* species plastomes. The phylogenetic analysis based on the complete plastomes strongly supported three main subclades in the *Maddenia* group of *Prunus*, while five subclades were recognized based on the nuclear tree. The phylogenetic network analysis detected six hybridization events. Integrating the nuclear and morphological evidence, we proposed to recognize five species within the *Maddenia* group, i.e., *Prunus fujianensis, P. himalayana, P. gongshanensis, P. hypoleuca*, and *P. hypoxantha*. Within this group, the first three species are well-supported, while the gene flow occurring throughout the *Maddenia* group seems to be especially frequent between *P. hypoleuca* and *P. hypoxantha*, eroding the barrier between them. The phylogenetic trees based on eight concatenated hypervariable regions had a similar topology with the complete plastomes, showing their potential as molecular markers and effective barcodes for further phylogeographic studies on *Maddenia*.

## Introduction

*Prunus* L. is a genus of more than 200 species, widely distributed in the temperate regions of the Northern Hemisphere and in the subtropics and tropics (Rehder, 1956; Yü et al., 1986; Lu et al., 2003; Hodel et al., 2021). Some taxa of *Prunus* (e.g., almonds, sweet cherries, peaches, and plums) are of significant economic value, and other species have also been used as ornamentals, timber, and medicine (Andro and Riffaud, 1995; Lee and Wen, 2001; Wen et al., 2008).

**Figure 1:**
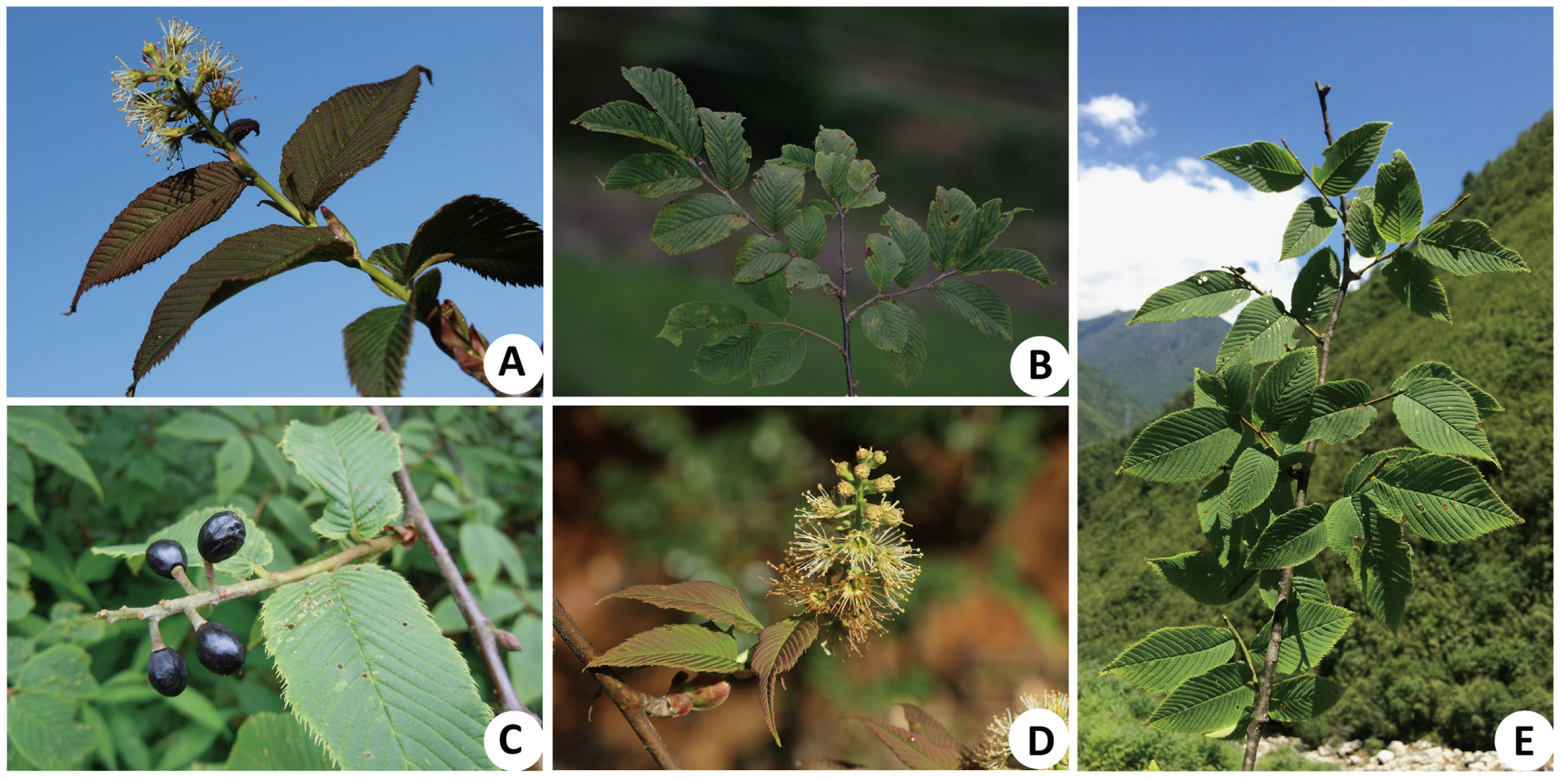
Morphological characteristics of *Maddenia* species. **(A)** *Prunus incisoserrata*; **(B,C)** *P. wilsonii*; **(B)** simple leaf and serrated leaf margin; **(C)** drupe; **(D,E)** *P*. *hypoxantha*; **(D)** racemose inflorescence; **(E)** simple leaf and serrated leaf margin.

*Maddenia* Hook. f. & Thoms was established as a genus by Hooker and Thomson (1854) and was later merged with *Prunus* by Chin et al. (2010) based on the phylogenetic analyses of nuclear and plastid DNA sequences. This provided strong support for the monophyly of *Maddenia* but it was resolved as nested within *Prunus*; these conclusions have also been supported by subsequent studies (Chin et al., 2014; Zhao et al., 2016, 2018; Wang et al., 2021). The *Maddenia* group of *Prunus* is characterized by its simple deciduous leaves with a serrate margin, terminal racemose inflorescences, 10 undifferentiated perianth parts at maturity, and drupe fruits (Figure 1; Focke, 1894; Yü et al., 1986; Lu et al., 2003; Kalkman, 2004; Wang et al., 2021). The group includes about 4–7 species endemic to East Asia, mainly distributed in the temperate regions of the Himalayas and eastern China, with China as its center of diversity, and one species in Bhutan, Nepal, and Sikkim of India (Rehder, 1956; Yü et al., 1986; Lu et al., 2003; Chin et al., 2010; Wen and Shi, 2012).

Within the *Maddenia* group, *Prunus himalayana* Hook. f. & Thomson was the first species described, followed by six other putative species (i.e., *P*. *hypoleuca* Koehne, *P*. *hypoxantha* Koehne, *P*. *wilsonii* Koehne, *P*. *fujianensis* Y. T. Chang, *P*. *incisoserrata* T. T. Yü & T. C. Ku, and *P*. *gongshanensis* J. Wen; Hooker and Thomson, 1854; Koehne, 1911; Chang, 1985; Yü et al., 1985; Lu et al., 2003; Wen and Shi, 2012). The species in this group were originally described based on morphological traits, especially the abaxial leaf pubescence (Yü et al., 1986; Lu et al., 2003; Wen and Shi, 2012). For example, *P. hypoxantha* and *P*. *wilsonii* were considered as two separate species based on the denser pubescence on the veins in *P. wilsonii*, and the two were also differentiated based on the size of their winter bud scales (Yü et al., 1986; Lu et al., 2003). However, Wen and Shi (2012) noted a continuous variation in the leaf pubescence between *P*. *hypoxantha* and *P*. *wilsonii*, and therefore treated the latter as a synonym of *P. hypoxantha*. This treatment was also supported by Shi et al. (2013), based on pollen morphology. Furthermore, the relationships among *P*. *fujianensis, P*. *hypoleuca*, and *P*. *incisoserrata* are poorly understood (Chang, 1985; Yü et al., 1986; Wen and Shi, 2012). *P. hypoleuca* was described based on its abaxially glabrous leaves, while *P*. *incisoserrata* and *P*. *fujianensis* both have pubescent abaxial leaf surfaces (Lu et al., 2003). Additionally, *P*. *incisoserrata* and *P*. *fujianensis* were recognized by some workers based on their leaf margin morphology (incised doubly serrate in the former vs. margin incised irregular serrate in the latter; Lu et al., 2003). However, in previous observations, we found that there was a continuous variation in the degree of abaxial pubescence as *P*. *hypoleuca* also has abaxially pubescent leaf blades and that there was a broad variation on the margin shape of *P*. *incisoserrata* and *P*. *fujianensis*, which greatly increased the difficulty in identifying them. In the latest revision of the *Maddenia* clade, Wen and Shi (2012) treated *P. fujianensis* and *P. incisoserrata* as synonyms of *P. hypoleuca* and they also recognized the former variety *P. himalaica* var. *glabrifolia* as a distinct species, *P. gongshanensis*.

It has been challenging to identify these species due to the existence of intermediate morphological features in the *Maddenia* clade. Traditional morphological methods alone cannot meet the needs for the species delimitation of the *Maddenia* group. With the rapid development of the phylogenetic analysis of *Prunus* s.l., the relationships within the *Prunus* and the *Maddenia* group have attracted new attention [see Chin et al. (2014)]. Yet to date, interspecific relationships within the *Maddenia* group are still unclear due to the limited taxon sampling and phylogenetically informative sites included in previous studies (Wen et al., 2008; Chin et al., 2010, 2014; Zhao et al., 2016, 2018).

Deoxyribonucleic acid (DNA) barcoding is an effective way to identify species by using a short DNA sequence (Kress et al., 2005; China Plant BOL Group, 2011; Li et al., 2015; Kress, 2017), however, DNA barcodes generally provide a limited number of informative sites among closely related taxa. As an alternative, genome skimming has been employed to generate complete chloroplast genomes (plastomes), an approach that has been dubbed as “super-barcoding” (Erickson et al., 2008; Yang et al., 2013; Li et al., 2015). The maternal inheritance and conservative genome structure of plastomes have rendered them essential markers in studying the evolutionary history of angiosperms (Gitzendanner et al., 2018; Do et al., 2020; Cai et al., 2021); noteworthy examples include the recent applications in Magnoliaceae (Wang et al., 2020), Rosaceae (Liu et al., 2019, 2020a,b), and Vitaceae (Wen et al., 2018). However, the uniparental inheritance of plastomes limits their power to fully elucidate the evolutionary histories of lineages with reticulate evolution, which has been proved to be very common in Rosaceae (Liu et al., 2020a,b; Hodel et al., 2021). In a case study on Vitaceae, Liu et al. (2021) proposed a new method for obtaining single-copy nuclear (SCN) genes from deep genome skimming data (minimum 10 × coverage for optimal performance), and this approach provided a good opportunity to infer phylogenetic relationships using the uniparentally inherited plastomes and the biparentally inherited nuclear genes. Additionally, with the rapid development of next-generation sequencing, it has been feasible to obtain genome skimming data efficiently and economically (Zimmer and Wen, 2015; Zhang N. et al., 2017).

In this study, we assembled 22 plastomes and captured 446 SCN genes from seven assumed species of *Maddenia* and five outgroup species in Amygdaloideae of Rosaceae (Xiang et al., 2016; Zhang S. D. et al., 2017). We also examined their morphological and micromorphological characteristics. We identified simple sequence repeats (SSRs) and repeat sequences from the plastomes of *Maddenia* clade species. Additionally, eight highly variable regions were determined from the plastomes. We aim to test the hypotheses on species delimitations and resolve the interspecific relationships within *Maddenia*, integrating the plastome, nuclear, and morphological evidence. We also aim to provide potential molecular markers and effective barcodes for further population-level studies on the *Maddenia* group.

## Materials and Methods

### Sampling, DNA Extraction, and Sequencing

For this study, 22 individuals were sampled, including 17 ingroup individuals from the *Maddenia* group and five outgroup species from the other clades of the Rosaceae subfamily, Amygdaloideae (Table 1), which includes three other species of *Prunus*. The 17 ingroup samples represented the taxonomic and geographic coverage of *Maddenia* (Yü et al., 1986; Lu et al., 2003). Total genomic DNAs were extracted from 15 mg of silica gel dried leaves using the Cetyltrimethylammonium Bromide (CTAB) method (Doyle and Doyle, 1987). The libraries were prepared at the Molecular Biology Experiment Center, Germplasm Bank of Wild Species in Southwest China using a NEBNext® Ultra™ II DNA Library Prep Kit (New England Biolabs, Ipswich, MA, USA). The paired-end (150 bp) sequencing of the DNA libraries was done on a HiSeq 2500 (Illumina, Inc., San Diego, CA, USA) platform in Beijing Genomics Institution (BGI) (Shenzhen, China), generating ~2 GB of raw data for each sample.

**Table 1:** Voucher information and GenBank accession numbers of sampled species.

**Taxon**	**Voucher**	**Location**	**Latitude (*N*)**	**Longitude (*E*)**	**Altitude (*m*)**	**Raw reads**	**Sequencing coverage**	**Mean coverage of plastomes(×)**	**GenBank accession number**	**SRA accession number**
*P. incisoserrata*	JR301	Pan tou lu, Qin xu xiang, Min xian County, Gansu, China	34°23′20.02″	103°55′44.28″	2,553	11,635,840	4.79	53.20	MN864470	SRR13868100
*P. incisoserrata*	JR334	Da dian, Taibai Mt., Shaanxi, China	34°3′39.17″	107°42′20.30″	2,005	14,681,382	6.05	27.20	MN864488	SRR13863260
*P. incisoserrata*	JR440	Ping he liang, Ning shan County, Shaanxi, China	33°28′59.66″	108°29′46.60″	2,324	20,462,458	8.41	184.73	MN864490	SRR13868097
*P. hypoleuca*	WX219	Dian bing chang, Taibai Mt., Shaanxi, China	34°4′51.51″	107°42′16.14″	1,310	19,977,616	7.30	70.30	MK911762	SRR12920653
*P. hypoleuca*	JR324	Shen nong jia Mt., Hubei, China	31°25′32.15″	110°16′51.25″	2,811	17,831,310	7.01	185.40	MN864482	SRR13863263
*P. hypoleuca*	JR336	Between Da dian and Ping an si, Taibai Mt, Shaanxi, China	34°1′19.28″	107°43′21.75″	2,665	17,536,240	6.85	99.17	MN864483	SRR13863262
*P. wilsonii*	WX202	Jin ding, Emei Mt., Sichuan, China	29°31′48.11″	103°20′13.01″	2,977	18,022,432	6.90	449.36	MK905682	SRR12920659
*P. wilsonii*	JR352	Pan tou lu, Qin xu xiang, Min xian County, Gansu, China	34°23′20.02″	103°55′44.28″	2,553	20,192,064	8.27	73.89	MN864491	SRR13863258
*P. wilsonii*	JR438	Ping he liang, Ning shan County, Sichuan, China	33°28′59.66″	108°29′46.60″	2,324	20,231,114	7.95	261.45	MN864493	SRR13868096
*P. hypoxantha*	JR372	Cai yuan zi cun, Kang ding County, Sichuan, China	30°3′40.51″	102°0′22.07″	2,498	20,459,760	8.39	35.00	MN864485	SRR13863257
*P. hypoxantha*	JR426	Tian men shi, Emei Mt., Sichuan, China	29°31′46.26″	103°20′7.75″	2,950	20,149,304	8.26	394.52	MN864486	SRR13868094
*P. himalayana*	JR377	A sang qiao, Ya dong County, Tibet, China	27°23′43.53″	88°58′32.80″	2,750	19,176,804	8.11	25.42	MN864480	SRR13863252
*P. himalayana*	JR385	Lin chang, Bo mi County, Tibet, China	29°51′36.25″	95°46′3.39″	2,612	20,387,188	8.27	83.04	MN864481	SRR13863251
*P. gongshanensis*	JR305	Dan zha cun, Hou qiao zhen, Teng chong County, Yunnan, China	25°32′55.89″	98°13′10.22″	2,600	18,584,668	7.67	12.02	MN864472	SRR13863265
*P. gongshanensis*	JR381	Lu ma deng xiang, Fu gong County, Yunnan, China	27°10′17.79″	98°45′36.96″	2,276	23,789,540	9.65	21.52	MN864477	SRR13863249
*P. fujianensis*	JR302	Huang gang Mt., Wuyi Mt., Fujian, China	27°52′18.53″	117°51′36.33″	2,140	21,000,492	8.27	318.80	MN864466	SRR13871574
*P. fujianensis*	JR386	Wuyi Mt., Qian shan County, Jiang xi, China	27°59′44.73″	117°48′52.92″	2,069	21,705,762	9.09	71.65	MN864475	SRR13863264
*P. discadenia*	WX203	Ning shan County, Shaanxi, China	33°23′54.95″	108°22′18.90″	2,158	75,563,260	29.52	2076.39	MK905683	SRR12927899
*P. serotina*	WX204	DC, USA	38°91′	−77°01′	120	18,698,840	7.20	780.63	MK905684	SRR12920648
*P. kansuensis*	WX207	Mei xian, Shaanxi, China	34°03′43.62″	107°45′34.68″	975	25,613,228	10.53	616.67	MK634746	SRR12920639
*Physocarpus amurensis*	WX230	Shaanxi, China	34°15′38.35″	108°04′9.03″	355	28,712,176	11.95	701.57	MK911770	SRR12920643
*Prinsepia uniflora*	WX231	Shaanxi, China	34°15′38.35″	108°04′9.03″	355	18,097,112	7.36	155.54	MK911771	SRR12920642

### Plastid Genome and Nuclear Ribosomal DNA (nrDNA) Assembly, Annotation, Visualization, and Phylogenetic Inference

The raw Illumina data were filtered for sequence quality using Trimmomatic v. 0.40 (Bolger et al., 2014) under default parameters. The filtered reads were assembled into plastome using the GetOrganelle pipeline (Jin et al., 2020). For a few accessions, Local Blast (Altschul et al., 1997) was used to align the contigs with the reference genomes (*Prunus armeniaca* (KY420025) and *P. salicina* (KY420002); Zhang N. et al., 2017; Zhang S. D. et al., 2017; Zhang X. et al., 2017). Finally, we concatenated each contig based on the orientation of the reference genome and obtained the consensus sequences through Geneious v.11.0.2 (Kearse et al., 2012). We annotated the assembled chloroplast genomes using Plastid Genome Annotator (PGA: Qu et al., 2019) and made minor manual adjustments using Geneious v.11.0.2. The transfer RNA (tRNA) genes were checked using tRNAscan-SE v.2.0 (Lowe and Chen, 2016). The circular plastid genome diagram was generated using the online OGDRAW (Lohse et al., 2013). The newly generated plastome sequence data of *Maddenia* and the other species of Rosaceae from this study have been submitted to GenBank (Table 1).

To obtain high-quality nuclear ribosomal DNA (nrDNA), including the Internal Transcribed Spacer (ITS) 1, 5.8S, and ITS2, a modified reference-based and *de novo* method (Zhang et al., 2015; Liu et al., 2019, 2020a,b) was employed for the assembly of the ITS sequences. The clean reads generated by Trimmomatic v. 0.40 (Bolger et al., 2014) were mapped to the reference sequence (*Prunus hypoleuca*: MH711078) using Bowtie2 v. 2.4.2 (Langmead and Salzberg, 2012), and then the draft sequence for each sample was generated. In addition, we conducted a *de novo* assembly using SPAdes v. 3.15.0 (Bankevich et al., 2012); the resulting scaffolds were used to correct the errors and ambiguities in the consensus sequences. Finally, we obtained high-quality nuclear ribosomal DNA (nrDNA) sequences for each sample using reference-based and *de novo* assembly methods.

We aligned the plastome and the ITS sequences using MAFFT (Katoh and Standley, 2013) using the software Geneious v.11.0.2 (Kearse et al., 2012). Based on maximum likelihood (ML) and Bayesian inference (BI) methods, we reconstructed the phylogeny of the *Maddenia* clade using the following nine datasets: (1) complete plastid genomes; (2) large-single-copy (LSC); (3) small-single-copy (SSC); (4) one inverted repeat (IR); (5) coding sequences (CDS); (6) non-coding region; (7) ITS; (8) concatenated sequence of *matK, rbcL*, and *trnH*-*psbA*; and (9) concatenated sequence of identified hypervariable regions. BI analyses were then conducted using MrBayes v.3.2 (Ronquist et al., 2012). The best-fitting models of nucleotide substitutions for BI analyses were determined based on the Akaike Information Criterion (AICc) through the CIPRES Science Gateway website (Miller et al., 2010). MrBayes was run for 10,000,000 generations, sampling every 1,000 generations. The first 25% of trees were discarded as a burn-in and the remaining trees were used to estimate the 50% majority-rule consensus tree and the Bayesian posterior probabilities (PP). For ML, all analyses were performed using the RAxML-HPC Black Box 8.2.12 (Stamatakis, 2014) with 10,000 bootstrap replicates and a GTR + G model at the CIPRES Science Gateway website (Miller et al., 2010).

### Plastome Comparisons and Identification of Hypervariable Regions

Gene rearrangement events within the *Maddenia* clade were detected using the Mauve v2.4.0 (Darling et al., 2010) software. We chose one *Maddenia* sequence from each species for plastome comparisons, which were performed online using mVISTA in Shuffle-LAGAN mode (Frazer et al., 2004). The reference sequence used was *P*. *wilsonii* WX202.

To identify the hypervariable regions, we used 22 plastomes to conduct the sliding window analysis in DnaSP v5 (Librado and Rozas, 2009) using a step size of 200 bp and a window length of 600 bp. We chose the sequences with relatively higher values of nucleotide diversity (Pi) as the hypervariable regions. The Pi refers to the difference of the chloroplast genome sequences among sequenced samples.

### Single-Copy Nuclear Marker Development, Gene Assembly, Alignment, and Phylogenetic Inference

As a part of the integrative systematic studies of *Prunus*, Hodel et al. (2021) identified 591 single-copy nuclear exons based on 17 transcriptomes of *Prunus*. Our genome skimming data were sequenced from the whole genomic DNA, which provided the opportunity to capture nuclear genes, including exon and intron sequences. We used three genomes of *Prunus* (*P. dulcis* (Mill.) D. A. Webb (https://www.ncbi.nlm.nih.gov/genome/10947), *P. mume* (Siebold) Sieb. et Zucc. (https://www.ncbi.nlm.nih.gov/genome/13911), and *P. persica* (L.) Batsch (https://www.ncbi.nlm.nih.gov/genome/388) as references to discover the corresponding complete genes (introns and exons) for the 591 exons. The resulting nuclear genes were used as references in the following gene assembly.

For assembling the SCN genes, we followed the pipelines of Liu et al. (2021). Briefly, the adapters and low-quality reads were trimmed using Trimmomatic v. 0.40 (Bolger et al., 2014), and the results were quality-checked using FastQC v. 0.11.9 (Andrews, 2018). The resulting clean reads were counted to calculate the sequencing coverage, assuming the genome size (352.9 Mb: Shirasawa et al., 2017) of *P. avium* (L.) L. HybPiper pipeline v. 1.3.1 (Johnson et al., 2016), with the default settings, was used to target the SCN genes; BWA v. 0.7.1 (Li and Durbin, 2009) was used to align and distribute the reads to the target genes; SPAdes v. 3.15.0 (Bankevich et al., 2012), with a coverage cutoff value of 5, was used to assemble the reads to the contigs; and Exonerate v. 2.2.0 (Slater and Birney, 2005) was used to align the assembled contigs to the target sequences and determine the exon-intron boundaries. To balance the quality and quantity of the captured SCN genes from the uneven sequencing coverage of genome skimming data (cf. Table 1), we used a relatively lower coverage cutoff for generating the contigs in SPAdes v. 3.15.0. Python and R scripts included in the HybPiper pipeline (Johnson et al., 2016) were used to retrieve the recovered gene sequences, and to summarize and visualize the recovery efficiency.

The sequences in each SCN gene were aligned using MAFFT v. 7.475 (Nakamura et al., 2018) with the settings: “–localpair –maxiterate1000.” Due to the variable sequencing depth in the genome skimming data, we employed three steps to remove the poorly aligned regions. In the first step, we used trimAL v. 1.2 (Capella-Gutiérrez et al., 2009) to trim the alignment of each SCN gene, in which all columns with gaps in more than 20% of the sequences or with a similarity score lower than 0.001 were removed. Considering the low-quality assembly in some regions, we used Spruceup (Borowiec, 2016) to discover, visualize, and remove the outlier sequences in the concatenated multiple sequence alignments with a window size of 50 and an overlap of 25. Because the Spruceup algorithm works better the more data it has, we concatenated all the SCN gene alignments using AMAS v. 1.0 (Borowiec, 2016) before running Spruceup, and we also used AMAS v. 1.0 (Borowiec, 2016) to split the processed/trimmed alignment back into single-locus alignments. The resulting alignments for each SCN gene were trimmed again using trimAL v. 1.2 (Capella-Gutiérrez et al., 2009) with the same parameters described above. At the third step, we excluded the sequences with <250 bp in each alignment using our customized python script (*exclude_short_sequences.py*), as the short sequences in each alignment have limited informative sites for the following coalescent-based species tree inference. Phylogenetic inference of the nuclear data of the *Maddenia* group was performed using both concatenated and coalescent-based methods. To reduce the effect of the missing data, gene alignments with at least 1,000 characters and 18 out of 22 taxa were retained. For the concatenation analysis, the best-fit partitioning schemes and nucleotide substitution models for the nuclear dataset were estimated using PartitionFinder2 (Stamatakis, 2006; Lanfear et al., 2016), under the corrected AICc and linked branch lengths, and with rcluster (Lanfear et al., 2014) algorithm options. The resulting scheme was then used to infer the ML trees using IQ-TREE 2 (Minh et al., 2020) and RAxML 8.2.12 (Stamatakis, 2014), respectively. To estimate the coalescent-based species tree, first, we inferred the individual ML gene trees using RAxML 8.2.12 (Stamatakis, 2014) with a GTRGAMMA model and 100 bootstrap replicates to assess the clade support, in which the low support branches (≤10) of gene trees were contracted by Newick Utilities (Junier and Zdobnov, 2010). The gene trees were then used to infer a species tree with ASTRAL-III v. 5.7.7 (Zhang et al., 2018) using local posterior probabilities (LPP; Sayyari and Mirarab, 2016) to assess clade support.

### Retrieving Standard DNA Barcodes

To determine if standard DNA barcodes can resolve the interspecific relationships of *Maddenia* species, we extracted the gene sequences of *matK, rbcL*, and *trnH*-*psbA* from annotated plastomes, and then concatenated them into a single aligned dataset in Geneious v.11.0.2 (Kearse et al., 2012).

### Phylogenetic Network Analyses

To explore the possibility of gene flow as a cause of discordance in the *Maddenia* group, we utilized 18 samples, including 17 *Maddenia* ingroups and one outgroup (*Prunus davidiana* (Carrière) Franch) for the phylogenetic network analyses. Species Networks applying Quartets (SNaQ: Solís-Lemus and Ané, 2016), as implemented in the Julia package PhyloNetworks (Solís-Lemus et al., 2017), was used to examine the contribution of the incomplete lineage sorting (ILS) and reticulation to the phylogenetic history of the *Maddenia* group. We used the ML tree inferred by RAxML for calculating the Concordance Factors (CFs), and the ASTRAL species tree was used as the input tree for SNaQ. We first tested the fit of the models, allowing from 0 to 8 reticulation events (h), and compared the models using their pseudolikelihood scores. For each number of hybrid nodes, we ran 50 SNaQ searches using the best topology from the previous run as a starting tree and retained the highest pseudolikelihood value. To distinguish the best fitting model, we used the log pseudolikelihood profile with h. A sharp improvement is expected until h reaches the best value and a slower, linear improvement thereafter. The best network was visualized in Julia using R.

### Characterization of SSRs and Repeat Sequences in Plastomes

We searched for SSRs in seven *Maddenia* species using MISA (Thiel et al., 2003) with the settings at 10, 5, 4, 3, 3, 3 repeat units for mono-, di-, tri-, tetra-, penta-, and hexanucleotide SSRs, respectively. Tandem Repeat Finder (Benson, 1999) was used to analyze the tandem repeat sequences with the default parameters. One inverted repeat sequence was removed before detecting large repeat sequences. We employed REPuter (Kurtz et al., 2001) to identify the large repeat sequences, including forward, reverse, complement, and palindromic repeats. The minimal repeat size and Hamming distance were set at 30 bp and 3, respectively.

### Morphological and Micromorphological Characteristics Detection

Images of mature leaves were taken with a Nikon SM225 Stereo microscope (Japan). To show the micromorphological traits, a scanning electron microscope (SEM) was used. The mature leaves were fixed in Formaldehyde-acetic acid-ethanol (FAA) (methanol: acetic acid: ethanol: water = 10:5:50:35), cut into small pieces, and washed in 70% alcohol. Then, they were dehydrated in an increasing alcohol series and iso-amyl acetate series. Afterward, the material was critical-point dried using liquid CO_2_ with a K850 critical-point dryer (Quorum). The leaf pieces were then mounted on aluminum stubs and sputter-coated with gold using a JS-1600 sputter coater (HTCY). Photos were taken with a Hitachi S-3400 SEM (Hitachi, Tokyo, Japan).

## Results

### Characteristics of *Maddenia* Plastomes

**Figure 2:**
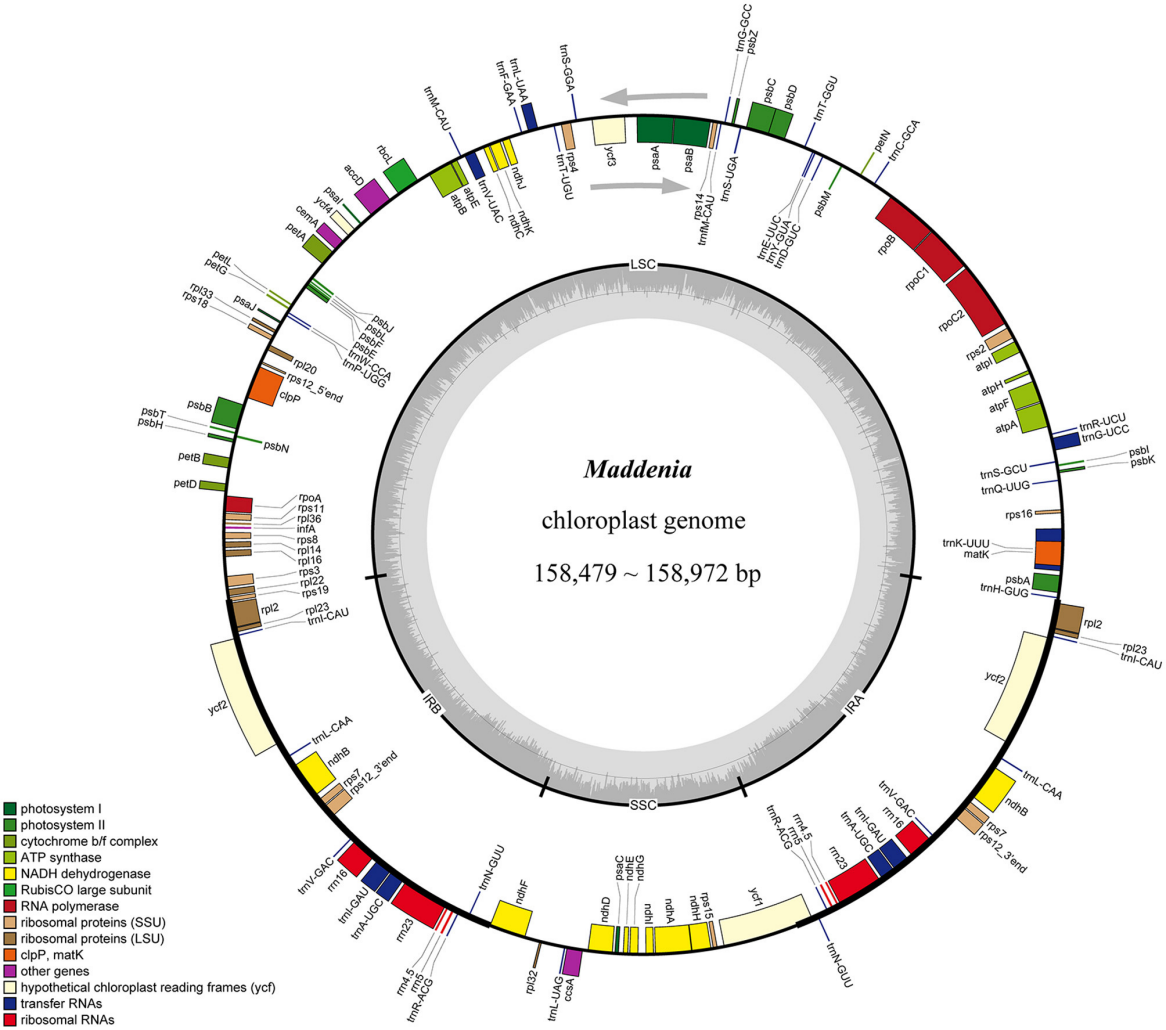
Gene map of *Maddenia* chloroplast genome. The two gray arrows indicate the direction of gene transcription. The dashed area in the inner circle indicates the GC content of the plastome. LSC, large-single-copy; SSC, small-single-copy; IR, inverted repeat.

We used genome skimming to generate DNA sequence data for 22 samples, including seven *Maddenia* species (17 individuals) and five outgroup species. The size of the *Maddenia* plastomes ranged from 158,479 to 158,972 bp in length. The plastomes of all the *Maddenia* species had a quadripartite structure (Figure 2), including a large single-copy region (LSC, 86,939–87,405 bp), a small single-copy region (SSC, 18,862–18,930 bp), and two inverted repeated regions (IRs, 26,292–26,363 bp) (Table 2). The total Guanine-cytosine (GC) content of all the *Maddenia* plastomes was 36.6%, but the GC content in IRs (42.5–42.6%) was higher than that in LSC (34.4%) and SSC (30.4–30.5%). All the *Maddenia* plastomes encoded 113 unique genes, including 79 protein-coding genes (CDS), four ribosomal RNAs (rRNAs), and 30 tRNAs. In addition, 17 genes were duplicated in the IRs, of which 6, 4, and 7 encoded proteins, rRNAs, and tRNAs, respectively (Table 2). In *Maddenia* plastomes, 14 unique genes had introns, of which two (*ycf3* and *clpP*) had two introns (Supplementary Table 1). The genome size, GC content, gene number, and order in all the *Maddenia* plastomes were relatively conserved in comparison to the outgroups (Table 2).

**Table 2:** Summary information for the plastome sequences of *Maddenia* species.

**Taxon**	**Number**	**Size (bp)**				**GC content (%)**				**Number of genes**			
		**Total**	**LSC**	**SSC**	**IR**	**Total**	**LSC**	**SSC**	**IR**	**Total**	**CDS**	**tRNA**	**rRNA**
*P. incisoserrata*	JR301	158,768	87,212	18,906	26,325	36.6	34.4	30.4	42.5	130 (17)	85 (6)	37 (7)	8 (4)
*P. incisoserrata*	JR334	158,882	87,347	18,885	26,325	36.6	34.4	30.5	42.5	130 (17)	85 (6)	37 (7)	8 (4)
*P. incisoserrata*	JR440	158,842	87,310	18,882	26,325	36.6	34.4	30.5	42.5	130 (17)	85 (6)	37 (7)	8 (4)
*P. hypoleuca*	WX219	158,873	87,338	18,885	26,325	36.6	34.4	30.5	42.5	130 (17)	85 (6)	37 (7)	8 (4)
*P. hypoleuca*	JR324	158,811	87,299	18,862	26,325	36.6	34.4	30.5	42.5	130 (17)	85 (6)	37 (7)	8 (4)
*P. hypoleuca*	JR336	158,881	87,346	18,885	26,325	36.6	34.4	30.5	42.5	130 (17)	85 (6)	37 (7)	8 (4)
*P. wilsonii*	WX202	158,673	87,134	18,889	26,325	36.6	34.4	30.5	42.5	130 (17)	85 (6)	37 (7)	8 (4)
*P. wilsonii*	JR352	158,751	87,198	18,903	26,325	36.6	34.4	30.4	42.5	130 (17)	85 (6)	37 (7)	8 (4)
*P. wilsonii*	JR438	158,908	87,373	18,885	26,325	36.6	34.4	30.5	42.5	130 (17)	85 (6)	37 (7)	8 (4)
*P. hypoxantha*	JR372	158,701	87,167	18,884	26,325	36.6	34.4	30.5	42.5	130 (17)	85 (6)	37 (7)	8 (4)
*P. hypoxantha*	JR426	158,760	87,206	18,894	26,330	36.6	34.4	30.5	42.5	130 (17)	85 (6)	37 (7)	8 (4)
*P. himalayana*	JR377	158,524	86,939	18,907	26,339	36.6	34.4	30.4	42.6	130 (17)	85 (6)	37 (7)	8 (4)
*P. himalayana*	JR385	158,827	87,208	18,893	26,363	36.6	34.4	30.4	42.5	130 (17)	85 (6)	37 (7)	8 (4)
*P. gongshanensis*	JR305	158,564	87,045	18,921	26,299	36.6	34.4	30.4	42.6	130 (17)	85 (6)	37 (7)	8 (4)
*P. gongshanensis*	JR381	158,479	86,965	18,930	26,292	36.6	34.4	30.4	42.6	130 (17)	85 (6)	37 (7)	8 (4)
*P. fujianensis*	JR302	158,972	87,405	18,873	26,347	36.6	34.4	30.5	42.5	130 (17)	85 (6)	37 (7)	8 (4)
*P. fujianensis*	JR386	158,954	87,390	18,870	26,347	36.6	34.4	30.5	42.5	130 (17)	85 (6)	37 (7)	8 (4)
*P. discadenia*	WX203	157,915	85,946	19,119	26,415	36.7	34.6	30.2	42.5	130 (17)	85 (6)	37 (7)	8 (4)
*P. serotina*	WX204	158,760	87,180	18,862	26,358	36.6	34.4	30.4	42.6	130 (17)	85 (6)	37 (7)	8 (4)
*P. kansuensis*	WX207	157,660	85,764	19,122	26,387	36.8	34.6	30.3	42.6	130 (17)	85 (6)	37 (7)	8 (4)
*Physocarpus amurensis*	WX230	159,110	87,563	18,827	26,360	36.4	34.1	30.0	42.6	130 (17)	85 (6)	37 (7)	8 (4)
*Prinsepia uniflora*	WX231	159,186	87,236	19,174	26,388	36.6	34.4	30.2	42.7	130 (17)	85 (6)	37 (7)	8 (4)

*The number in braces means duplicated gene number in the IR region*.

### Plastome Comparisons

**Figure 3:**
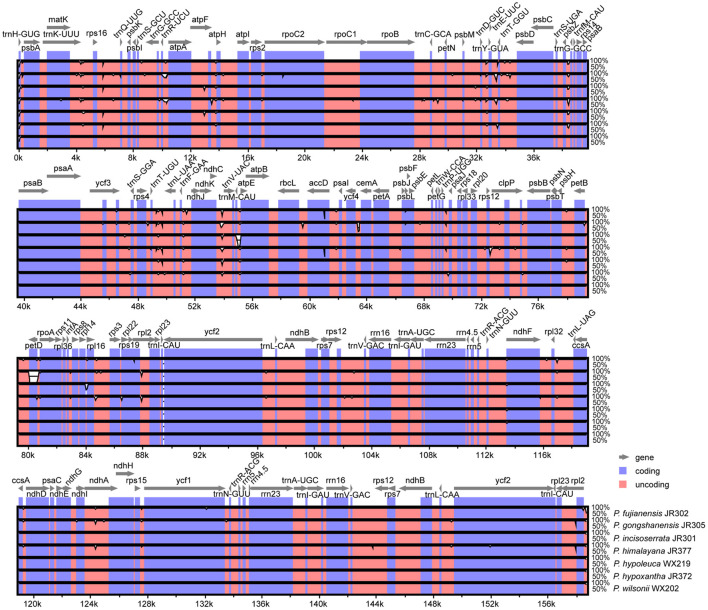
Visualization of alignment of the seven *Maddenia* chloroplast genome sequences using mVISTA. *P*. *wilsonii* WX202 was used as a reference sequence. Blue represents coding regions, and pink represents non-coding regions.

Overall, *Maddenia* plastomes showed high sequence similarity, and non-coding regions had more divergence than coding regions (Figure 3). In the Mauve analysis, no rearrangement event was detected among the *Maddenia* plastomes (Supplementary Figure 1).

Nucleotide substitution and sequence distance were used to compare the difference of plastomes between the seven *Maddenia* species. Across all individuals, the number of nucleotide substitutions was 0–266 bp and the pairwise sequence distance percentage among the whole plastome sequences was 0–0.00169. The sequence differences between *P*. *fujianensis, P*. *himalayana*, and *P*. *gongshanensis* were much higher than those between *P*. *hypoleuca, P*. *hypoxantha, P*. *incisoserrata*, and *P*. *wilsonii* (Table 3).

**Table 3:** Numbers of nucleotide substitutions and sequence distance in *Maddenia* plastomes.

	***P*. *fujianensis* JR302**	***P*. *fujianensis* JR386**	***P. gongshanensis* JR305**	***P. gongshanensis* JR381**	***P. himalayana* JR377**	***P. himalayana* JR385**	***P. hypoleuca* WX219**	***P. hypoleuca* JR324**	***P. hypoleuca* JR336**	***P. hypoxantha* JR372**	***P. hypoxantha* JR426**	***P. incisoserrata* JR301**	***P. incisoserrata* JR334**	***P. incisoserrata* JR440**	***P. wilsonii* WX202**	***P. wilsonii* JR352**	***P. wilsonii* JR438**
*P*. *fujianensis* JR302		0.00000629	0.00140	0.00145	0.00163	0.00105	0.00093	0.00095	0.00095	0.00094	0.00107	0.00088	0.00095	0.00095	0.00103	0.00086	0.00096
*P. fujianensis* JR386	1		0.00139	0.00145	0.00162	0.00102	0.00093	0.00095	0.00094	0.00093	0.00107	0.00087	0.00094	0.00093	0.00102	0.00087	0.00093
*P. gongshanensis* JR305	222	220		0.00020	0.00160	0.00090	0.00128	0.00139	0.00130	0.00142	0.00142	0.00130	0.00130	0.00093	0.00144	0.00130	0.00135
*P. gongshanensis* JR381	230	230	32		0.00169	0.00101	0.00138	0.00143	0.00134	0.00145	0.00147	0.00134	0.00134	0.00138	0.00149	0.00134	0.00139
*P. himalayana* JR377	257	256	253	266		0.00123	0.00156	0.00163	0.00152	0.00161	0.00168	0.00154	0.00152	0.00154	0.00164	0.00154	0.00154
*P. himalayana* JR385	166	161	143	160	194		0.00100	0.00110	0.00099	0.00101	0.00109	0.00092	0.00099	0.00094	0.00105	0.00090	0.00097
*P. hypoleuca* WX219	148	147	202	218	246	158		0.00024	0	0.00023	0.00037	0.00015	0	0.00017	0.00103	0.00014	0.00018
*P. hypoleuca* JR324	151	151	220	226	258	174	38		0.00025	0.00027	0.00046	0.00023	0.00025	0.00020	0.00037	0.00022	0.00021
*P. hypoleuca* JR336	150	149	205	212	240	157	0	39		0.00023	0.00037	0.00015	0	0.00018	0.00025	0.00014	0.00019
*P. hypoxantha* JR372	149	147	225	229	254	160	36	43	36		0.00045	0.00021	0.00023	0.00018	0.00036	0.00014	0.00019
*P. hypoxantha* JR426	170	169	224	233	266	173	58	73	58	72		0.00037	0.00037	0.00040	0.00030	0.00036	0.00042
*P. incisoserrata* JR301	140	138	206	212	244	145	24	37	24	34	58		0.00015	0.00016	0.00026	0	0.00017
*P. incisoserrata* JR334	150	149	205	212	240	157	0	39	0	36	58	24		0.00018	0.00025	0.00014	0.00019
*P. incisoserrata* JR440	150	147	147	218	243	149	27	31	28	28	63	25	28		0.00030	0.00015	0.00001
*P. wilsonii* WX202	163	162	228	235	259	167	163	58	40	57	48	41	40	47		0.00025	0.00030
*P. wilsonii* JR352	137	138	205	211	243	143	23	35	23	23	57	0	23	24	40		0.00016
*P. wilsonii* JR438	152	148	213	220	244	153	29	33	30	30	66	27	30	1	48	25	

*The upper triangle shows the number of nucleotide substitutions and the lower triangle indicates the number of sequence distances in the Maddenia plastomes*.

### IRs Expansion and Contraction

**Figure 4:**
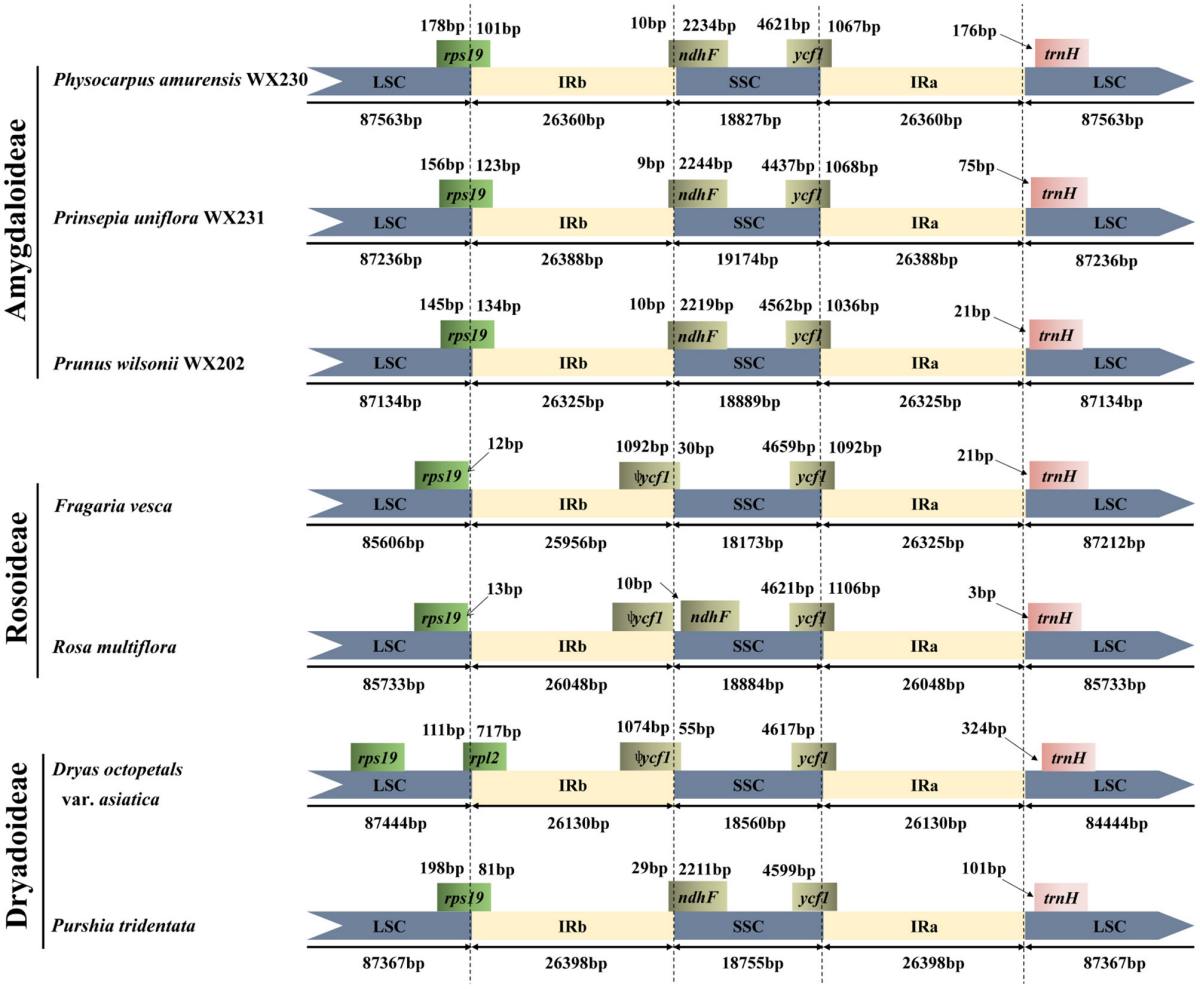
Comparison of the LSC, IRs, and SSC border regions of *Maddenia* group and other Rosaceae plastomes.

Given that there were no significant differences among the *Maddenia* plastomes (Supplementary Figures 1, 2), *P. wilsonii* WX202 in *Maddenia* was chosen to conduct border comparisons. Six Rosaceae species, i.e., *Physocarpus amurensis* (Maxim.) Maxim. WX230 and *Prinsepia uniflora* Batalin WX231 from Amygdaloideae; *Rosa multiflora* Thunb. (NC_039989) and *Fragaria vesca* L. (NC_015206) from Rosoideae; *Dryas octopetala* var. *Asiatica* (Nakai) Nakai (KY420029) and *Purshia tridentata* (Pursh) DC. (KY420000) from Dryadoideae, were compared to *P. wilsonii* WX202. Variation was detected in the expansion and contraction of IR regions (Figure 4). The LSC/IRb borders of Amygdaloideae species and *Purshia tridentata*were located in the *rps19* gene, which extended 81–134 bp into the IRb. In Rosoideae species, the LSC/IRb borders were in the intergenic spacers, and the intact *rps19* gene in the LSC contracted 12–13 bp from the LSC/IRb border. In addition, the SSC/IRs borders were in the *ndhF*/ψ*ycf1* and *ycf1* genes except for the *Rosa multiflora* (only in *ycf1* gene). The IRb/SSC border was located in the pseudogene *ycf1* for *Fragaria vesca* and *Dryas octopetala* var. *asiatica*, and between the pseudogene *ycf1* and *ndhF* gene for *Rosa multiflora*. The *ndhF* gene extended 9–29 bp into the IRb in the Amygdaloideae species and *Purshia tridentata* but was completely located in the SSC for Rosoideae species and *Dryas octopetala* var. *asiatica*. The SSC/IRa border was located in the *ycf1* gene across the Rosaceae species. The gene *trnH* in the LSC contracted 3–324 bp from the border region of IRa/LSC.

### Identification of Hypervariable Regions

**Figure 5:**
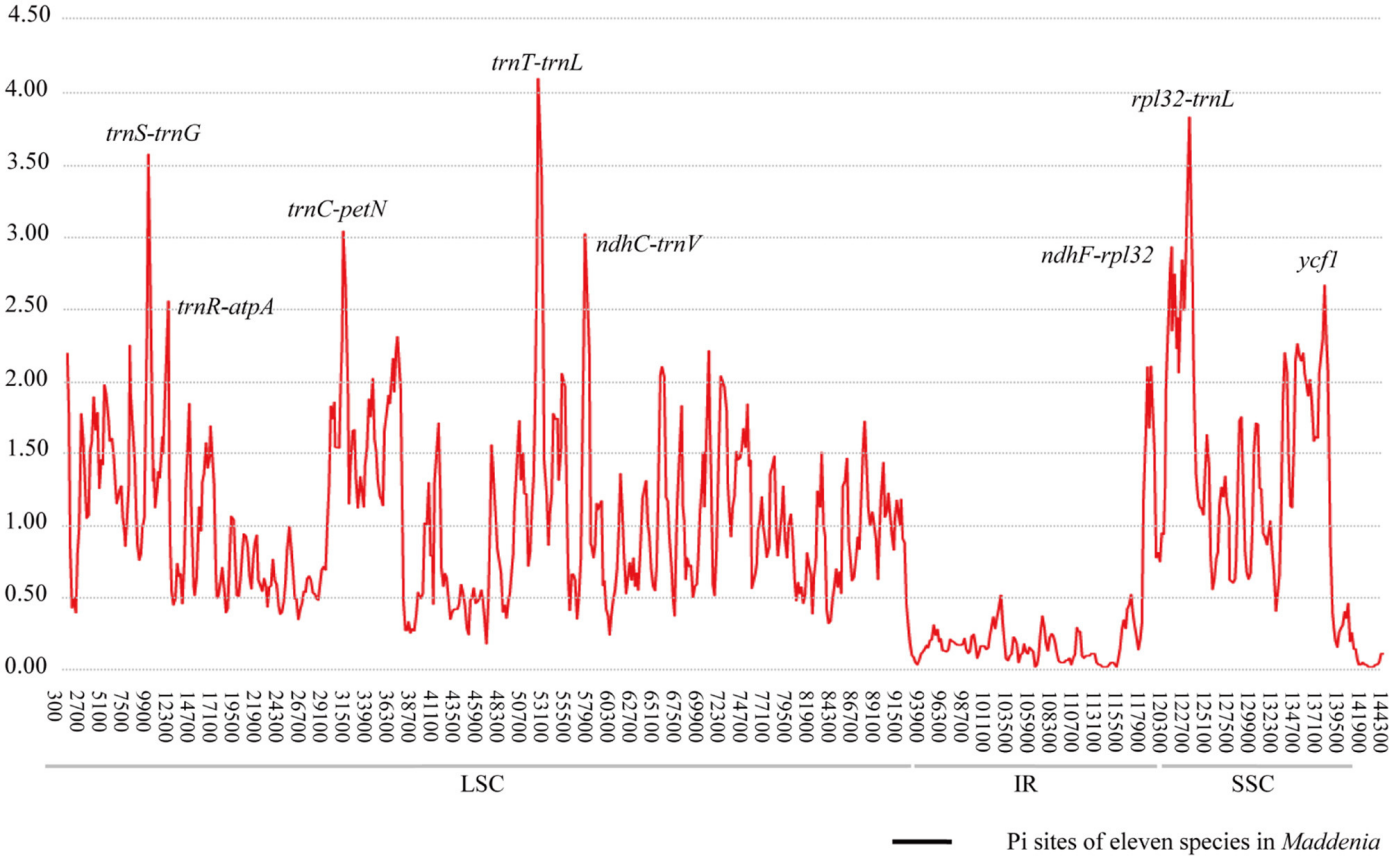
Sliding window analysis of the plastomes of samples.

The Pi values were used to determine hypervariable regions. The result showed that the Pi values in IRs were less than those in LSC and SSC. We chose the regions with relatively higher Pi values as hypervariable regions. A total of eight hypervariable regions were identified, including seven intergenic spacer regions (*trnS–trnG, trnR–atpA, trnC–petN, trnT–trnL, ndhC–trnV, ndhF–rpl32*, and *rpl32–trnL*) and one protein-coding region (*ycf1*). These sequences were all located in two single-copy regions and none in IR regions (Figure 5). The Pi value of eight hypervariable regions ranged from 0.01619–0.03251 (Table 4).

**Table 4:** Sequence characteristics of eight high variable regions among 22 plastomes.

**Region**	**Aligned length**	**Variable sites**		**Indels**		**Nucleotide diversity (Pi)**
		**No**.	**%**	**No**.	**Length range**	
*trnS*-*trnG*	711	57	8.01	32	1–301	0.02656
*trnR*-*atpA*	582	27	3.63	44	1–146	0.03251
*trnC*-*petN*	1,075	155	14.41	36	1–44	0.02258
*trnT*-*trnL*	1,367	135	9.87	77	1–114	0.02468
*ndhC*-*trnV*	772	83	10.75	49	1–160	0.02798
*ndhF*-*rpl32*	1,241	172	13.85	60	1–174	0.02746
*rpl32*-*trnL*	1,774	163	9.18	78	1–263	0.02570
*ycf1*	5,730	702	12.25	36	1–30	0.01619

### Repeat Analyses

**Figure 6:**
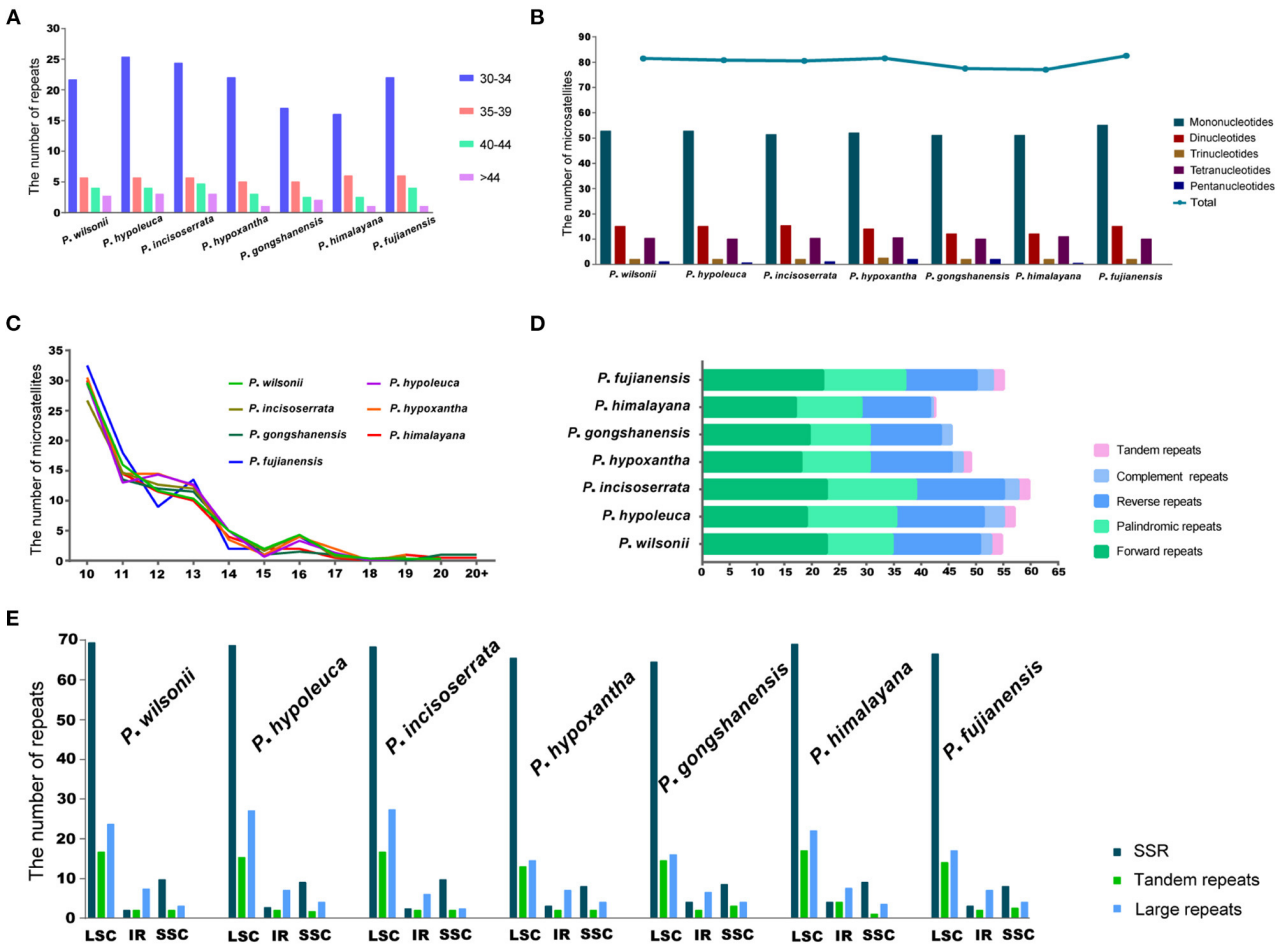
Analyses of simple sequence repeats (SSRs) and repeated sequences in plastomes of *Maddenia* species. **(A)** Frequency of four repeats by length; **(B)** Number of five repeat types; **(C)** Frequency of microsatellites by length; **(D)** Numbers of five different types of repeats; **(E)** Number of all repeats by location.

A total of 558 SSRs were identified in seven *Maddenia* species, including mono-, di-, tri-, tetra-, and pentanucleotide, but there was no hexanucleotide in all plastomes (Figure 6B). *P*. *fujianensis* showed the most SSRs, followed by *P*. *hypoxantha, P*. *wilsonii, P*. *hypoleuca, P*. *incisoserrata*, and *P*. *himalayana*, while the SSR numbers of *P*. *gongshanensis* were the least (Figure 6B). There were many SSR motif types in the *Maddenia* plastomes, but most of them had few SSRs and only two types (A/T and AT/TA) contained more SSRs (Supplementary Figure 3A). The lengths of SSRs ranged from 10 to 24 bp (Figure 6C).

In *Maddenia* plastomes, the repeat sequences included forward, reverse, palindromic, complement, and tandem repeats. *P*. *incisoserrata* contained the most repeat sequences, *P*. *himalayana* had the least, and *P*. *gongshanensis* had no tandem repeats (Figure 6D). The most common type of repeat sequences by length was 30–34 bp (Figure 6A).

Most of the SSRs and repeat sequences were located in the LSC, followed by the SSC and IRs (Figure 6E). In addition, repeat sequences were mainly distributed in the intergenic spacers (IGS), but some were also found in the CDS and intron regions (Supplementary Figure 3B).

### Phylogenetic Analysis

**Figure 7:**
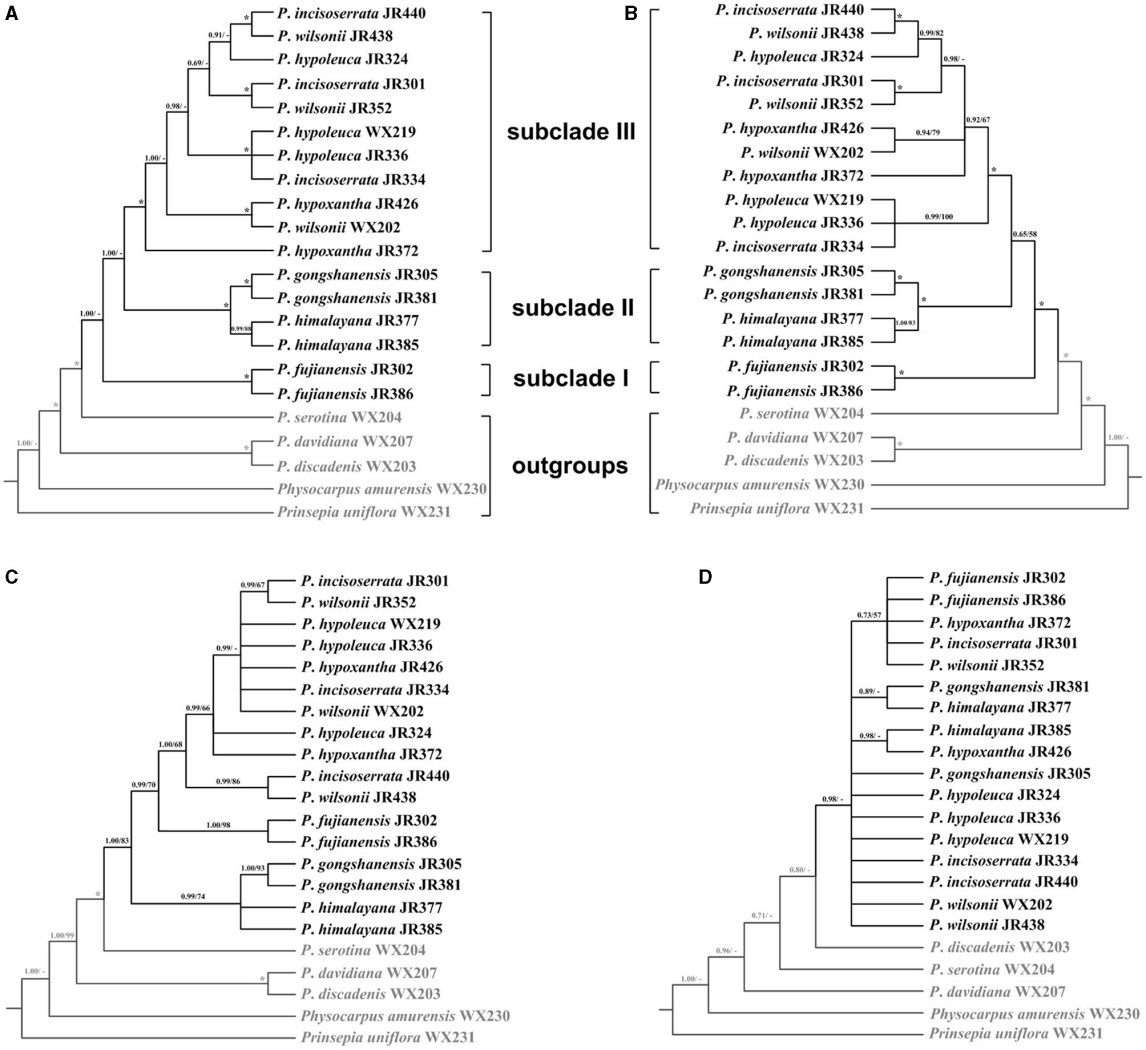
Phylogenetic relationships of the *Maddenia* group of *Prunus* inferred from Bayesian inference (BI) and maximum likelihood (ML) based on four datasets. **(A)** complete plastomes; **(B)** hypervariable regions; **(C)** *rbcL*+*matK*+*trnH-psbA*; **(D)** internal transcribed spacer (ITS) sequence. The support values above the branches show PP (posterior probability) / BS (bootstrap support), and asterisks indicate 1.00/100%. Dashes represent incongruences of BI and ML trees.

To resolve the phylogenetic relationship of the *Maddenia* clade, different trees were reconstructed based on complete plastomes and SCN genes. For plastome data, all the trees had identical topology except SSC and IR (Figure 7A and Supplementary Figure 4). In the rest of this section, the tree based on complete plastomes will be used to discuss the phylogenetic relationships of the *Maddenia* group, which was monophyletic and was separated into three subclades with high support values. Subclade I only include one species (*P*. *fujianensis*) from Fujian Province of eastern China. Subclade II is sister to subclade III, and together they are both sister to subclade I with a posterior probability of 1.00 Subclade II consists of *P*. *gongshanensis* from Yunnan Province (China) and *P*. *himalayana* from Tibet (China) and the adjacent Himalayan region. For the individuals sampled, the two species are reciprocally monophyletic. Subclade III is composed of four former species with a posterior probability of 1.00 and bootstrap value of 100%, in which samples from the same geographical position were grouped, although the four species were each not clearly identified.

To save costs of sequencing for further investigations on *Maddenia*, we also tried to explore four standard DNA barcodes to identify *Maddenia* species. Concatenated *rbcL, matK*, and *trnH-psbA* and ITS datasets were used to construct the phylogenetic trees of *Maddenia*, respectively. The trees constructed by the standard DNA barcodes were not congruent with those reconstructed using the complete plastomes (Figures 7C,D). In the phylogenic tree based on concatenated *rbcL, matK*, and *trnH-psbA*, although the *Maddenia* species formed a clade, *P*. *fujianensis* was sister to subclade III rather than to the remaining *Maddenia* species. In addition, the two *P*. *himalayana* individuals did not group, and subclade III exhibited more polytomies than the tree based on complete plastomes. For the tree based on ITS sequences, there were many polytomies and the interspecific relationships within *Maddenia* were poorly resolved.

Eight concatenated hypervariable regions (“specific barcodes”; see Discussion) were also employed to reconstruct the phylogenetic relationship of the *Maddenia* clade. We found that the topology based on the hypervariable regions was similar to that of complete plastomes, though there were lower support values at some branches (Figure 7B).

**Figure 8:**
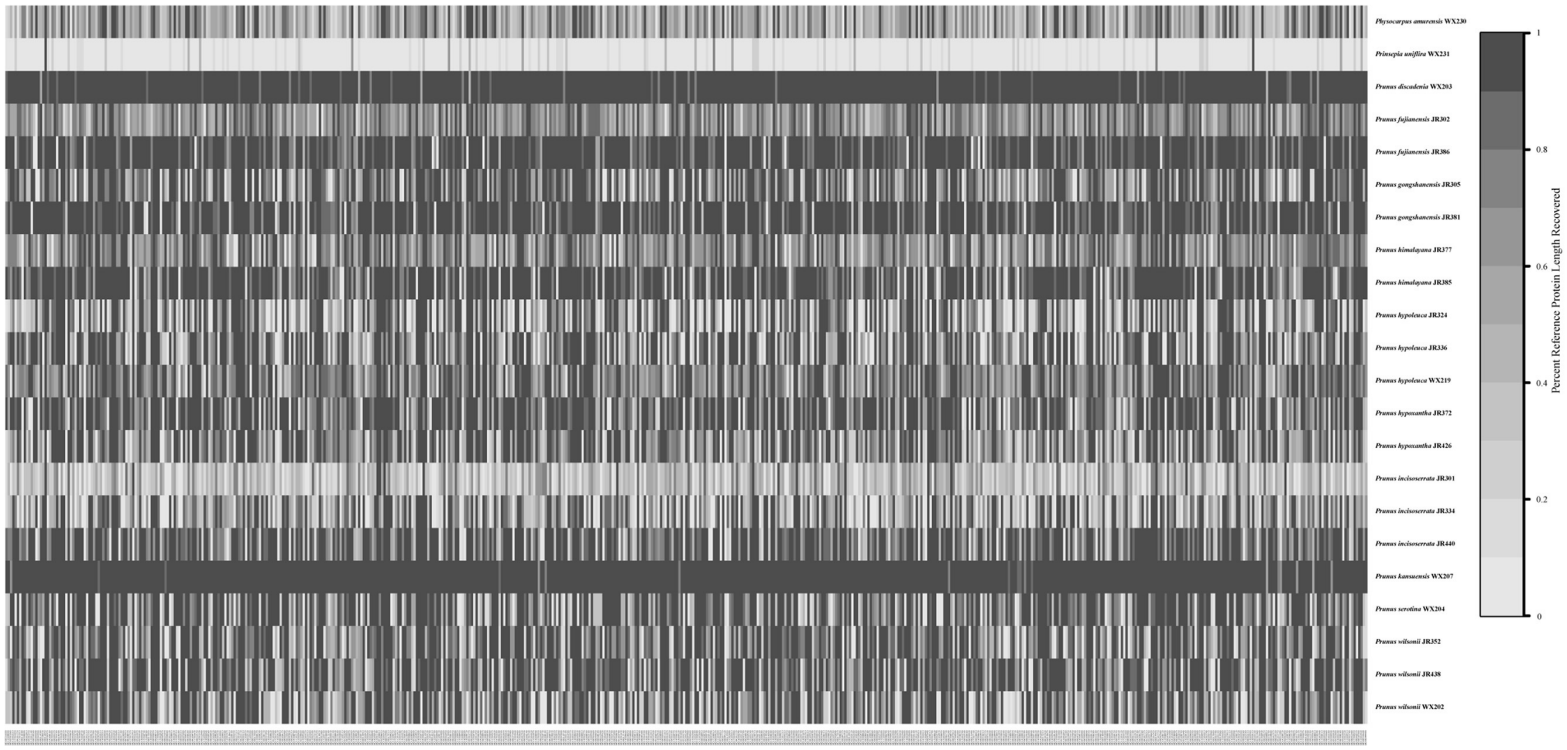
Heat map showing recovery efficiency for 591 genes enriched in the *Maddenia* group recovered by HybPiper. Each column is a gene, and each row is one sample. The shade of gray in the cell is determined by the length of sequence recovered by the pipeline, divided by the length of the reference gene (maximum of 1).

**Figure 9:**
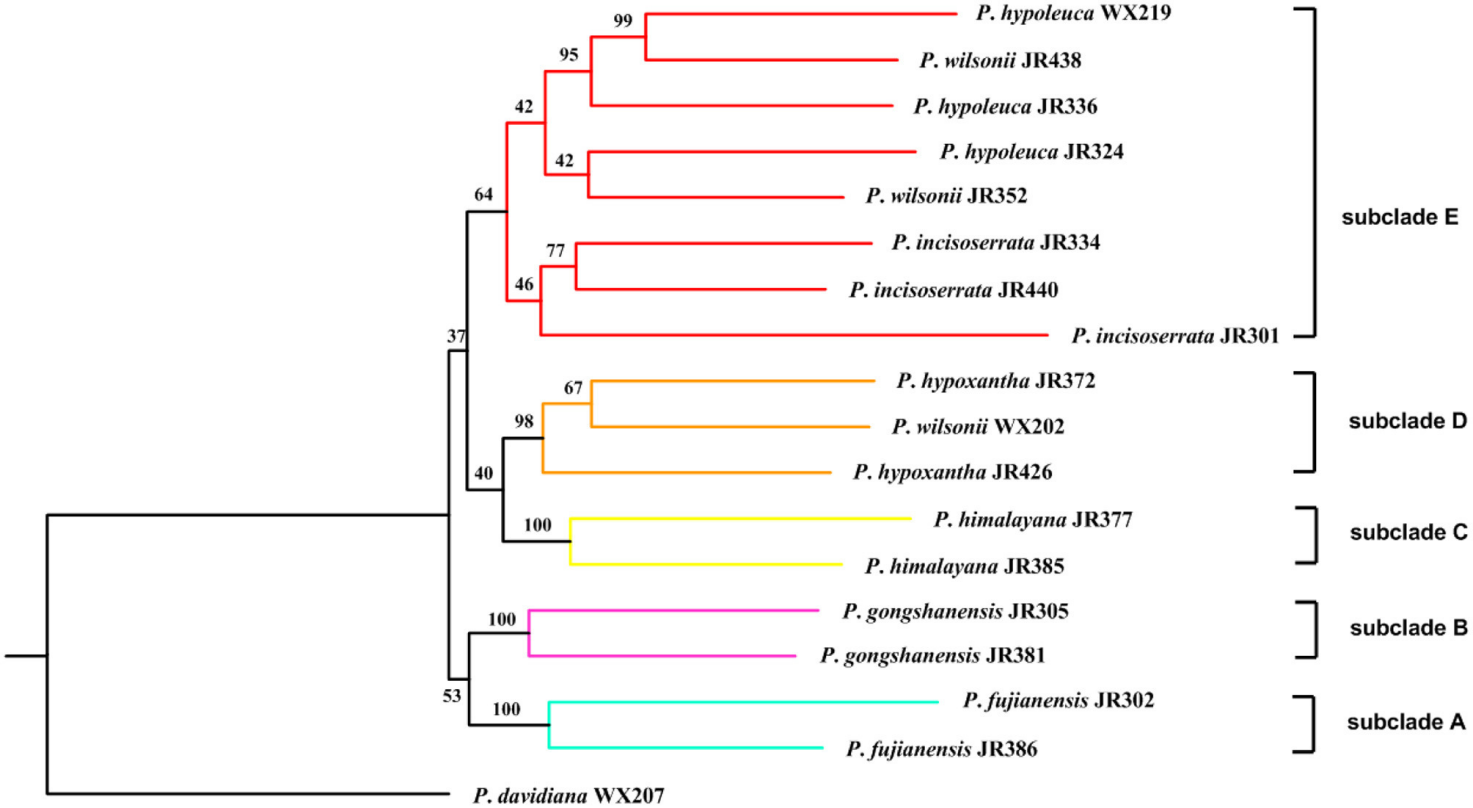
Maximum likelihood inferred from 446 single-copy nuclear (SCN) genes of *Maddenia* group of *Prunus*. The number above branch indicated bootstrap support from the IQ-TREE.

The recovery efficiency of each SCN gene is shown in Figure 8. The quality of the nuclear genes recovered was relatively high. In total, we got 446 SCN genes from raw data. We also filtered out genes with <80% samples, leaving 413 SCN genes with more than 600 bp in length. For the tree generated by 446 SCN genes data, the *Maddenia* clade was monophyletic but its deep nodes were not resolved well (Figure 9). It was obvious that there were five subclades in the *Maddenia* internal clade according to their geographic positions (e.g., Fujian Province, Yunnan Province, Tibet, Sichuan Province, and Qingling Mountain). Subclades A, B, and C comprise *P*. *fujianensis, P*. *gongshanensis*, and *P*. *himalayana*, respectively. The monophyly of each of the three species was well-supported, which was congruent with that of 413 SCN genes (Supplementary Figure 5). Subclade D consists of samples of *P*. *hypoxantha* and one individual of *P*. *wilsonii*. Subclade E contains *P*. *hypoleuca, P*. *incisoserrata*, and two individuals of *P*. *wilsonii*. However, these four species are not identified clearly.

### Phylogenetic Network Analyses of Nuclear Data With SNaQ

**Figure 10:**
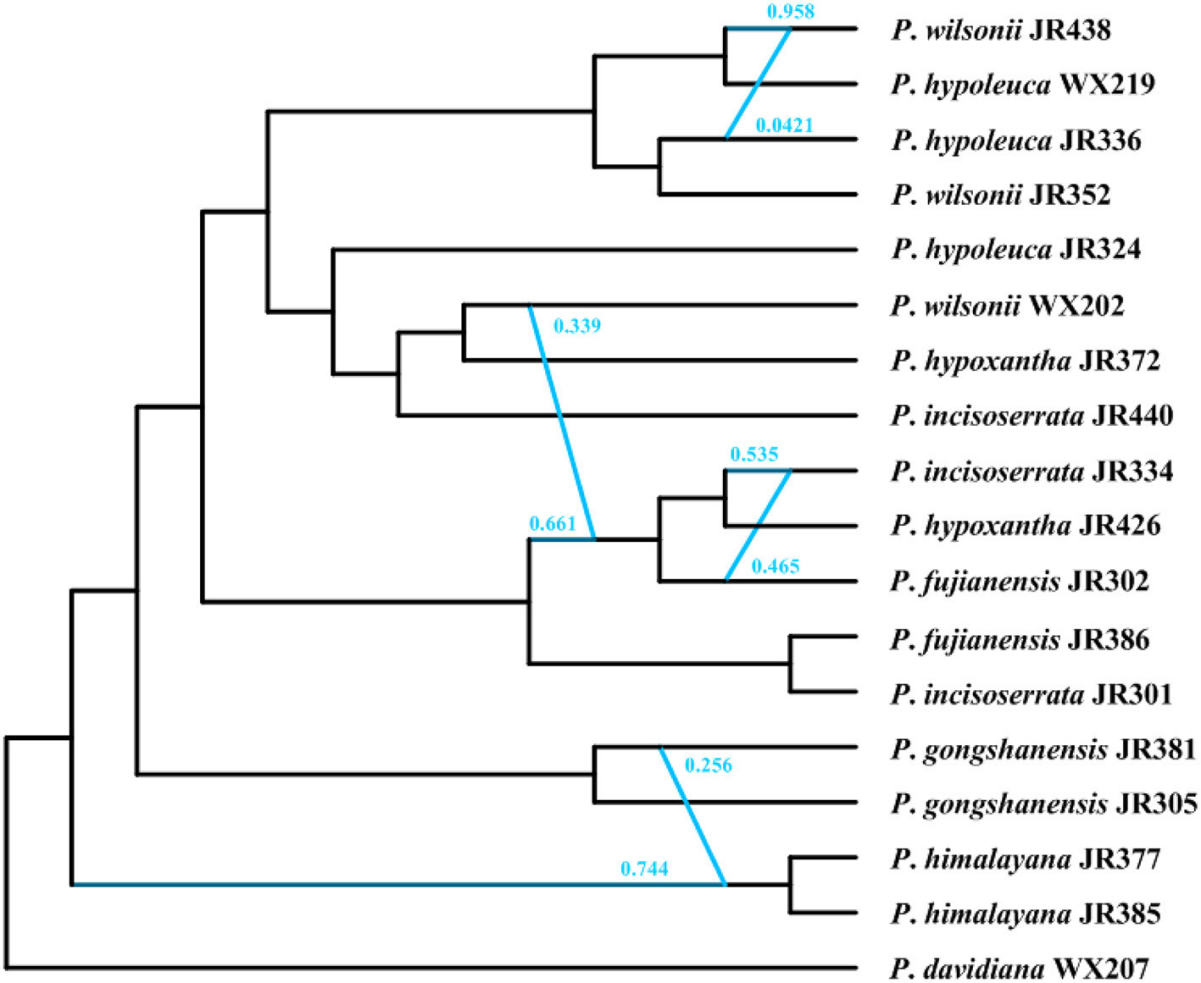
The phylogenetic network inferred in the SNaQ/PhyloNetworks analysis. The blue line indicated the hybridization events in the network.

The optimal *h*_*max*_ value inferred in the SNaQ was six, which has the highest pseudolikelihood network score (−676.478, Supplementary Figure 6). The phylogenetic network analyses showed that there were widespread hybridization events within the *Maddenia* group (Figure 10). The two *P. himalayana* individuals were 74.4% sister to each other and 25.6% sister to *P*. *gongshanensis* JR381. The group (*P*. *fujianensis* JR302+*P*. *hypoxantha* JR426+ *P*. *incisoserrata* JR334) was 66.1% sister to (*P*. *fujianensis* JR386+ *P*. *incisoserrata* JR301) and 33.9% sister to *P*. *wilsonii* WX202. *P*. *incisoserrata* JR334 was 53.5% sister to *P*. *hypoxantha* JR426 and 46.5% sister to *P*. *fujianensis* JR302. *P*. *wilsonii* JR438 was 95.8% sister to *P*. *hypoleuca* WX219 and 4.21% sister to *P*. *hypoleuca* JR336.

### Morphological and Micromorphological Traits

**Figure 11:**
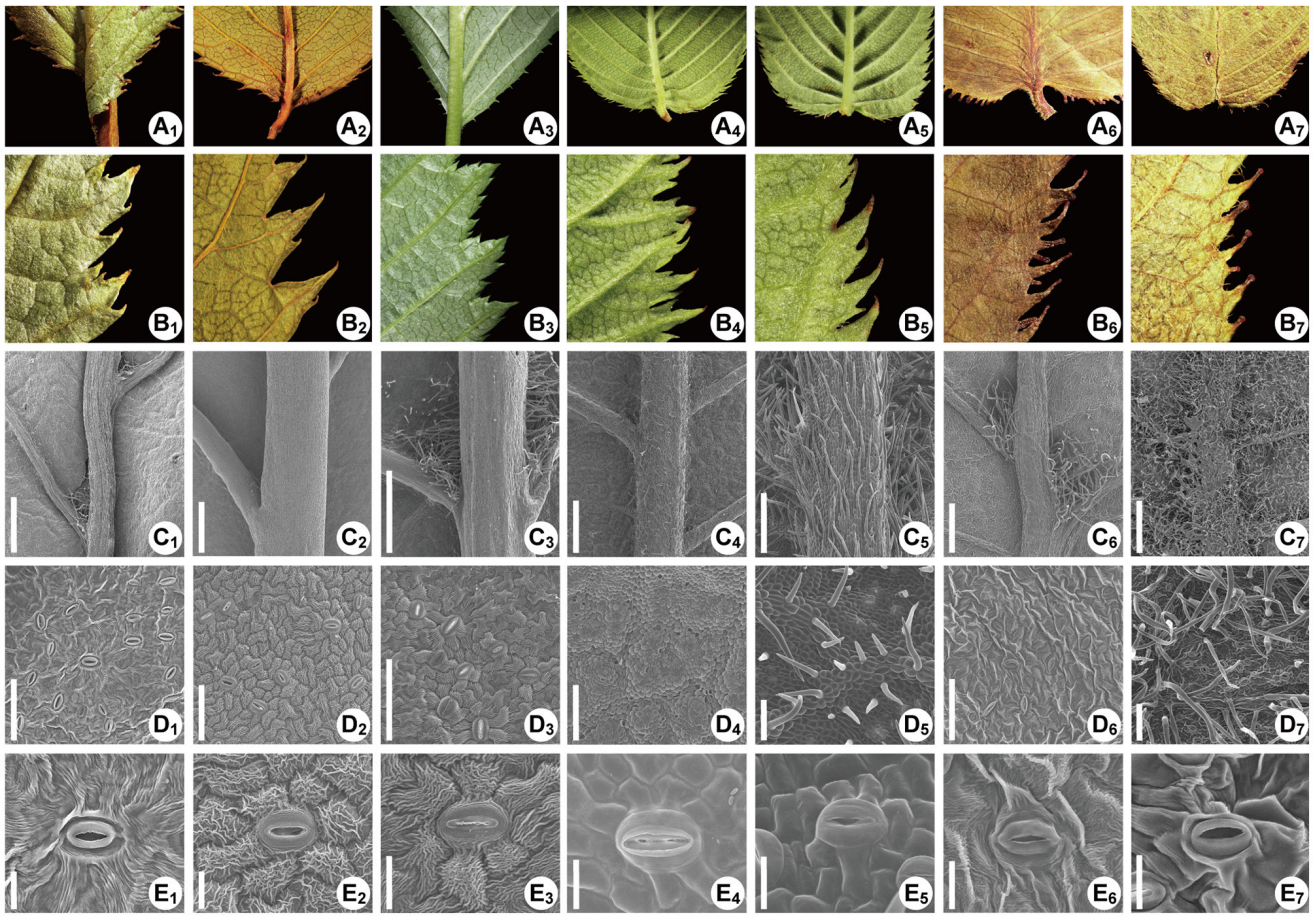
The leaf morphology of the *Maddenia* species. The species are *Prunus fujianensis, Prunus hypoleuca, P*. *incisoserrata, Prunus hypoxantha, P*. *wilsonii, Prunus gongshanensis*, and *Prunus himalayana*, respectively in each row from left to right. **(A**_**1**_**-A**_**7**_**)** Leave bases, showing the glandular teeth; **(B**_**1**_**-B**_**7**_**)** Partial views of leaf margins, showing the shapes of teeth and glandular teeth; **(C**_**1**_**-C**_**7**_**)** Abaxial leaves, showing the pubescence distribution; **(D**_**1**_**-D**_**7**_**)** Detailed view of the pubescence on intercostal areas; **(E**_**1**_**-E**_**7**_**)** Stomata and guard cells, showing the ornamentation.

The mature leaves of the *Maddenia* species are green to deep green adaxially. The shapes of the leaves and leaf bases are multiple (Figure 11A_1−7_). The leaf margins are serrulate, irregularly serrate, or doubly serrate. A few glandular teeth were found at the leaf bases of *P. hypoleuca, P. incisoserrata, P. wilsonii, P. hypoxantha*, and *P. fujianensis* (Figure 11B_1−5_), while many glandular teeth grow at the lower margins of *P. gongshanensis* (Figure 11A_6_). For *P. himalayana*, leaf margins have less glandular on the foliage branch while, in the reproductive branch, the glandular teeth are distributed abundantly near the bases (Figure 11B_7_).

The most notable morphological character differentiating species in *Maddenia* is the hair distribution on the abaxial leaf surface. In *P. fujianensis*, few hairs were found at the axils between midvein and secondary veins, and there is no hair on the intercostal area (Figure 11C_1_,D_1_). The intercostal area of *P. hypoleuca* and *P. incisoserrata* are also glabrous, but the distribution pattern of hairs at the axils shows high diversity (Figure 11C_2_,_3_,D_2_,_3_). There were no hairs or only a few hairs growing on the bases of secondary veins, or there was a cluster of hairs growing on the axil. Such three situations could be found on a single blade of the leaf. In *P. wilsonii* and *P. hypoxantha* hairs grow all along the veins, but the hairs are present on veinlets in the intercostal area of *P. wilsonii*, which distinguishes it from *P. hypoxantha* (Figure 11C_4_,_5_,D_4_,_5_). Hairs were also observed at the axils of *P. gongshanensis*, and sometimes there are a few hairs on the midvein at the base (Figure 11C_6_,D_6_). Leaves of *P. himalayana* are densely pubescent on the abaxial side (Figure 11C_7_,D_7_).

In all seven species, stomata are found only on the abaxial surface, and each of them consists of a pair of guard cells encircled by several other cells (Figure 11E_1−7_). Distinct circular ornamentations were found on the cell wall of guard cells in *P. hypoleuca* and *P. incisoserrata* (Figure 11E_2_,_3_). In the other species, such ornamentations are relatively obscure or nearly inexistent (Figure 11E_1_,_4−7_).

## Discussion

### Comparative Plastomes of *Maddenia*

All sequenced *Maddenia* plastomes share a typical quadripartite structure, which is similar to most photosynthetic angiosperms (Jansen and Ruhlman, 2012; Abdullah et al., 2019; Xu et al., 2019). However, the loss of one complete IR region also occurred in some taxa, such as the inverted-repeat-lacking clade of Fabaceae (Wang et al., 2017), *Erodium* of Geraniaceae (Guisinger et al., 2011), and *Carnegiea* of Cactaceae (Sanderson et al., 2015). In addition, the GC content in the IRs was higher than that in LSC and SSC, which is due to the presence of rRNA genes with high GC content (Kim and Lee, 2004). The conserved genome size, GC content, and gene number of *Maddenia* plastomes resemble other Amygdaloideae species (Wang et al., 2013; Kim et al., 2018). Although gene rearrangement events have been reported in some genera of other families, such as *Lasthenia* of Asteraceae (Walker et al., 2014), *Anemone* of Ranunculaceae (Liu et al., 2018), and *Passiflora* of Passifloraceae (Rabah et al., 2018), we observed no such events in *Maddenia* plastomes (Supplementary Figure 1).

The expansion and contraction of the IR region have an impact on the plastome size to some extent (Jansen and Ruhlman, 2012). Expansion events caused several genes in SC regions to enter the IR region. However, small IR expansions and contractions have a much higher frequency than large ones in seed plants (Goulding et al., 1996; Downie and Jansen, 2015). For *Maddenia*, no significant variation and slight IR expansions and contractions exist in every border of plastomes (Supplementary Figure 2), demonstrating their conserved traits. Nevertheless, compared with other Rosaceae species, we observe that Rosoideae plastomes have a partial *rps19* gene in the LSC region but an intact *rps19* gene in the LSC of Amygdaloideae. Variations in the location of the *rps19* gene were also documented in other Rosaceae species (e.g., Wang et al., 2013; Kim et al., 2018). However, our results indicate that the location of the *rps19* gene is not useful in distinguishing the three subfamilies, since the two Dryadoideae plastomes we analyzed showed two different locations of the *rps19* gene: one matching Amygdaoideae and the other matching a member of Rosoideae.

The SSRs are effective molecular markers to population genetic and phylogenetic studies in plants (Powell et al., 1995; Doorduin et al., 2011; Zhang X. et al., 2017; Sun et al., 2020). A total of 558 SSRs were identified in seven *Maddenia* species (Figure 6). The SSRs motif type (A/T) was quite common and most of SSRs were located in the intergenic spacers, which were similar to other Rosaceae species (Wang et al., 2013). In addition, previous studies supported that the region rich in A/T had the most repeats and indels (Cai et al., 2008). Thus, *Maddenia* SSRs could be further utilized for population genetics research in the future.

### Phylogenetic Analyses and Implications for Species Delimitation

Our results provided strong support for the monophyly of the *Maddenia* clade based on both plastome and nuclear datasets. The phylogenetic trees based on complete plastomes and nuclear datasets divided *Maddenia* into three and five major subclades, respectively. Subclade I in the plastome tree (= subclade A in the nuclear tree) included one species (*P*. *fujianensis*) only distributed at Wuyi Mountain of Fujian Province in Southeast of China. *Maddenia fujianensis* has been treated as a synonym of *P. hypoleuca*, for the differentiating characters between them are continuous (Wen and Shi, 2012). *P. fujianensis* was, however, sister to all remaining species of the *Maddenia* group based on plastome data. Even though this relationship was not congruent with that of SCN genes (Figure 9), the monophyly of *P. fujianensis* was well-supported. The SNaQ analyses suggested one hybridization event between *P*. *fujianensis* JR302 and *P*. *incisoserrata* JR334 (Figure 10). Therefore, it is likely that *P. fujianensis* may be a cryptic species, even though it is morphologically and micromorphologically similar to *P. hypoleuca* (Figure 11). More attention should be focused on the origin of *P*. *fujianensis* in our future studies, sampling *P. hypoleuca* broadly across its entire distribution range.

Subclade II in the plastome tree consists of *P*. *gongshanensis* and *P*. *himalayana* (= subclades B and C, respectively, in the nuclear tree) distributed in Yunnan Province (Northwest Hengduan Mountains) and Southeast Tibet, respectively. *P*. *gongshanensis* is characterized by subcordate to cordate leaf bases and glabrous leaf surfaces. *P*. *himalayana* stands out by its abaxially dense pubescent leaf blade. Although these two species can be identified based on both molecular, morphological, and micromorphological evidence, one hybridization event between them was detected in the SNaQ analyses (Figures 10, 11).

Subclade III in the plastome tree (comprising subclades D and E in the nuclear tree) is composed of the remaining four species, but their relationships were not resolved. Interestingly, most different species from the same geographical area were grouped, such as *P*. *hypoxantha* JR426 and *P*. *wilsonii* WX202 from the Emei Mountain of Sichuan Province and *P*. *hypoxantha* JR372 from Kangding of Sichuan Province, and all others from the Qinling Mountain region. Meanwhile, the nuclear tree showed that these four species were divided into two groups according to their geographical distribution. Therefore, we assume that *Maddenia* subclade III might represent two species, i.e. *P. hypoleuca* and *P. hypoxantha*, which is congruent with the treatment of Wen and Shi (2012). *P*. *incisoserrata* may best be merged with *P*. *hypoleuca* because they cannot be reliably distinguished by either molecular or morphological evidence (Figure 11). Although *P*. *hypoxantha* and *P*. *wilsonii* can be identified, to some extent, by the distribution of pubescence on the abaxial leaf surface (i.e., pubescence only on veins vs. denser pubescence on veins (Figure 11), we conclude that the latter should be treated as a synonym of the former due to the unsolved relationship between them in the various phylogenetic trees based on our results. Moreover, the sequence differences among species of subclade III of chloroplast tree are minimal (Table 3). Gene flow might be widespread within these two species as detected by the SNaQ analyses (Figure 10). Future studies should aim to explore the speciation history of subclade III using a broader populational sampling scheme.

### On Specific Barcoding of *Maddenia*

Considering the limitations of the standard DNA barcodes and the higher cost of super barcoding, an alternative approach known as “specific barcoding” was proposed, which combined the advantages of the other two (Li et al., 2015). Specific barcoding uses the sequences in target plastomes with high mutation rates. Compared to standard DNA barcodes, specific barcoding is more applicable for differentiation among closely related taxa (Li et al., 2015). We detected eight hypervariable regions among 22 individual plastomes, most of which are located in intergenic spacers (Figure 5). This result as well as those from mVISTA, SSRs, and repeat sequence analyses support that the intergenic spacers harbor the highest levels of variation in plastomes. High variability regions in the intergenic spacer have been reported in other studies and shown excellent discriminating ability, such as *Echinacea* of Asteraceae (Zhang N. et al., 2017), *Rhodiola* of Crassulaceae (Zhao et al., 2019), and *Pulsatilla* of Ranunculaceae (Li et al., 2020). Therefore, developing specific barcodes in the intergenic spacers is well-founded and should provide a reliable approach to assess the phylogenetic relationships and identification among *Maddenia* species.

The tree estimated from the specific barcoding regions (Figure 7B) had a similar topology with that of the complete chloroplast genomes. However, the sister relationship between subclades II and III was relatively weakly supported (posterior probability of 0.65 and bootstrap support of 58%). At the same time, the gene flow between the species of subclade III within *Maddenia* is active. The utility of these barcodes from chloroplast genomes would be limited for this group. More sampling in population-scale and high-throughput sequencing nuclear data such as RAD or whole-genome resequencing are needed to further explore the relationships of the species of subclade III.

## Data Availability Statement

The datasets presented in this study can be found in online repositories. The names of the repository/repositories and accession number(s) can be found in the article.

## Author Contributions

LZ and JW planned and designed the research. NS, B-BL, J-RW, CR, and R-CT performed the experiments and analyzed the data. NS, B-BL, J-RW, R-CT, CR, Z-YC, LZ, DP, and JW wrote the manuscript. All authors approved the final manuscript.

## Funding

This project was supported by the National Natural Science Foundation of China (Nos. 32170381, 31770200, 32000163, and 31300158) and the Chinese Universities Scientific Fund (No. 2452020179).

## Conflict of Interest

The authors declare that the research was conducted in the absence of any commercial or financial relationships that could be construed as a potential conflict of interest.

## Publisher's Note

All claims expressed in this article are solely those of the authors and do not necessarily represent those of their affiliated organizations, or those of the publisher, the editors and the reviewers. Any product that may be evaluated in this article, or claim that may be made by its manufacturer, is not guaranteed or endorsed by the publisher.

## Supplementary Material

Supplementary Figure 1 Structural variation between plastomes of *Maddenia* and outgroups revealed by Mauve.

Supplementary Figure 2 Comparison of the large-single-copy (LSC), inverted repeats (IRs), and small-single-copy (SSC) border regions of *Maddenia* plastomes.

Supplementary Figure 3 Microsatellites number of different base compositions and distribution of all repeated sequences. **(A)** Frequency of microsatellites by base composition; **(B)** Number of all repeats by location. CDS, coding sequences; IGS, intergenic spacers.

Supplementary Figure 4 Phylogenetic relationships of *Maddenia* inferred from Bayesian inference (BI) and maximum likelihood (ML) based on six datasets. **(A)** complete plastomes; **(B)** coding regions; **(C)** LSC region; **(D)** non-coding regions; **(E)** SSC region; **(F)** IR region. The support values above the branches show PP (posterior probability) / BS (bootstrap support), and asterisks indicate 1.00/100%. Dashes represent incongruences of BI and ML trees.

Supplementary Figure 5 Maximum likelihood inferred from 413 single-copy nuclear (SCN) genes of *Maddenia* group of *Prunus*. The number above branch indicated bootstrap support from the IQ-TREE.

Supplementary Figure 6 The pseudolikelihood network score from the Species Networks applying Quartets (SNaQ) analysis for each of the maximum number of hybridizations allowed (*h*_*max*_).

Supplementary Table 1 Genes present in the 17 complete *Maddenia* chloroplast genomes.
