# Supplementary figures and images for: On the Species Delimitation of the Maddenia Group of Prunus (Rosaceae): Evidence From Plastome and Nuclear Sequences and Morphology
Source: Front Plant Sci. 2021 Oct 11;12:743643. doi: 10.3389/fpls.2021.743643 (PMC8542774; doi:10.3389/fpls.2021.743643)

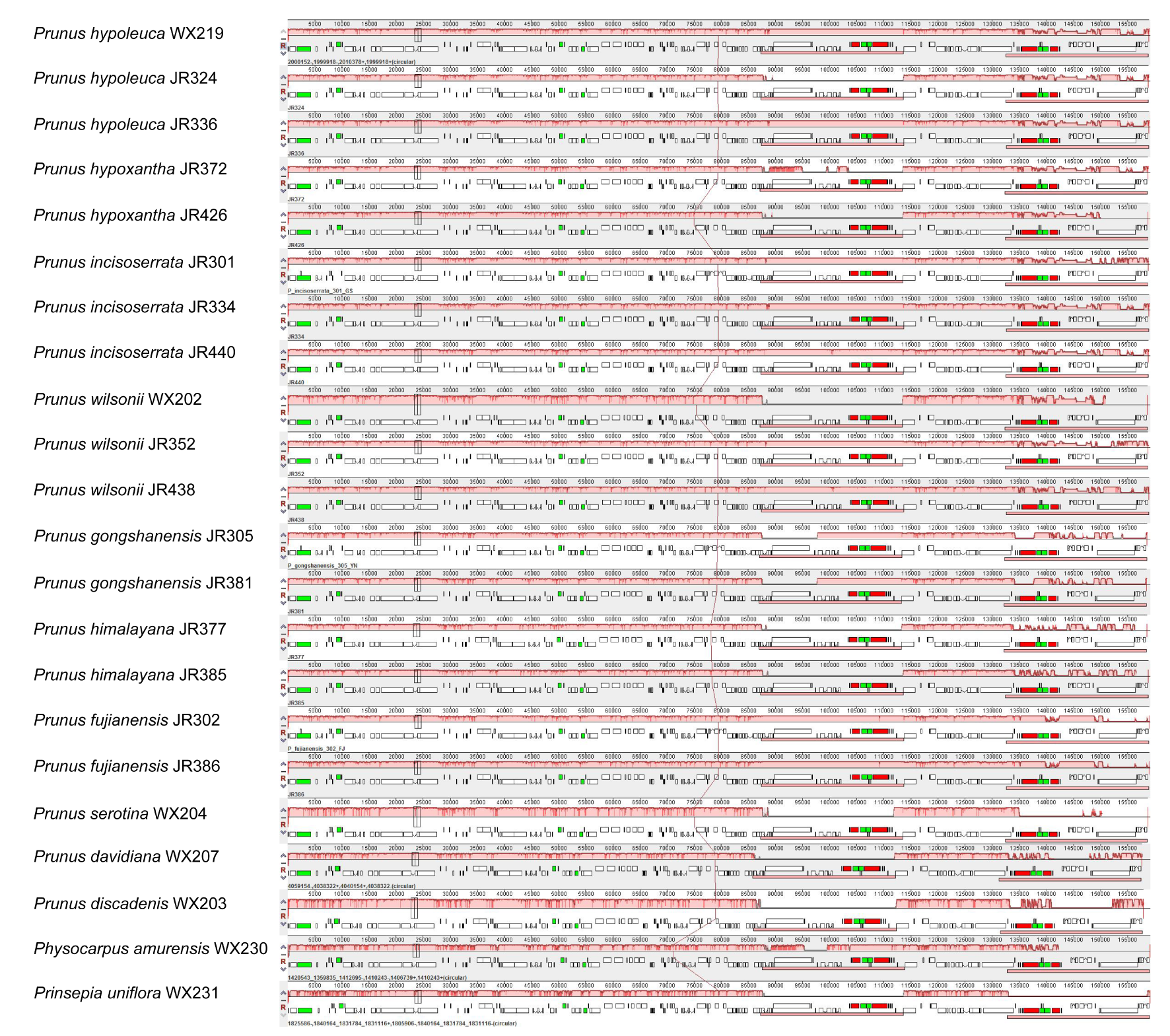

Supplement: Supplementary Figure 1 — Structural variation between plastomes of Maddenia and outgroups revealed by Mauve. [file Image_1.TIF]

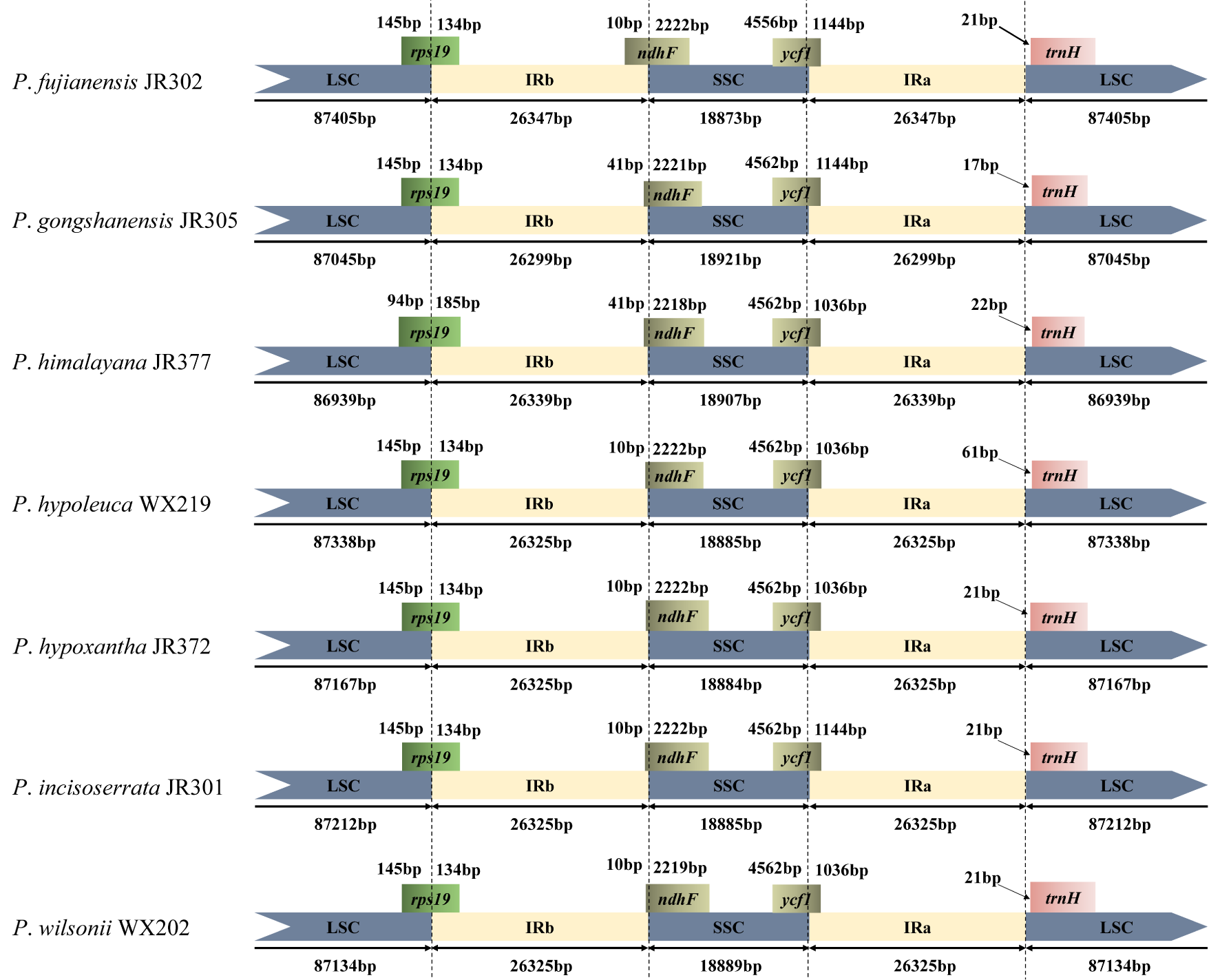

Supplement: Supplementary Figure 2 — Comparison of the large-single-copy (LSC), inverted repeats (IRs), and small-single-copy (SSC) border regions of Maddenia plastomes. [file Image_2.TIF]

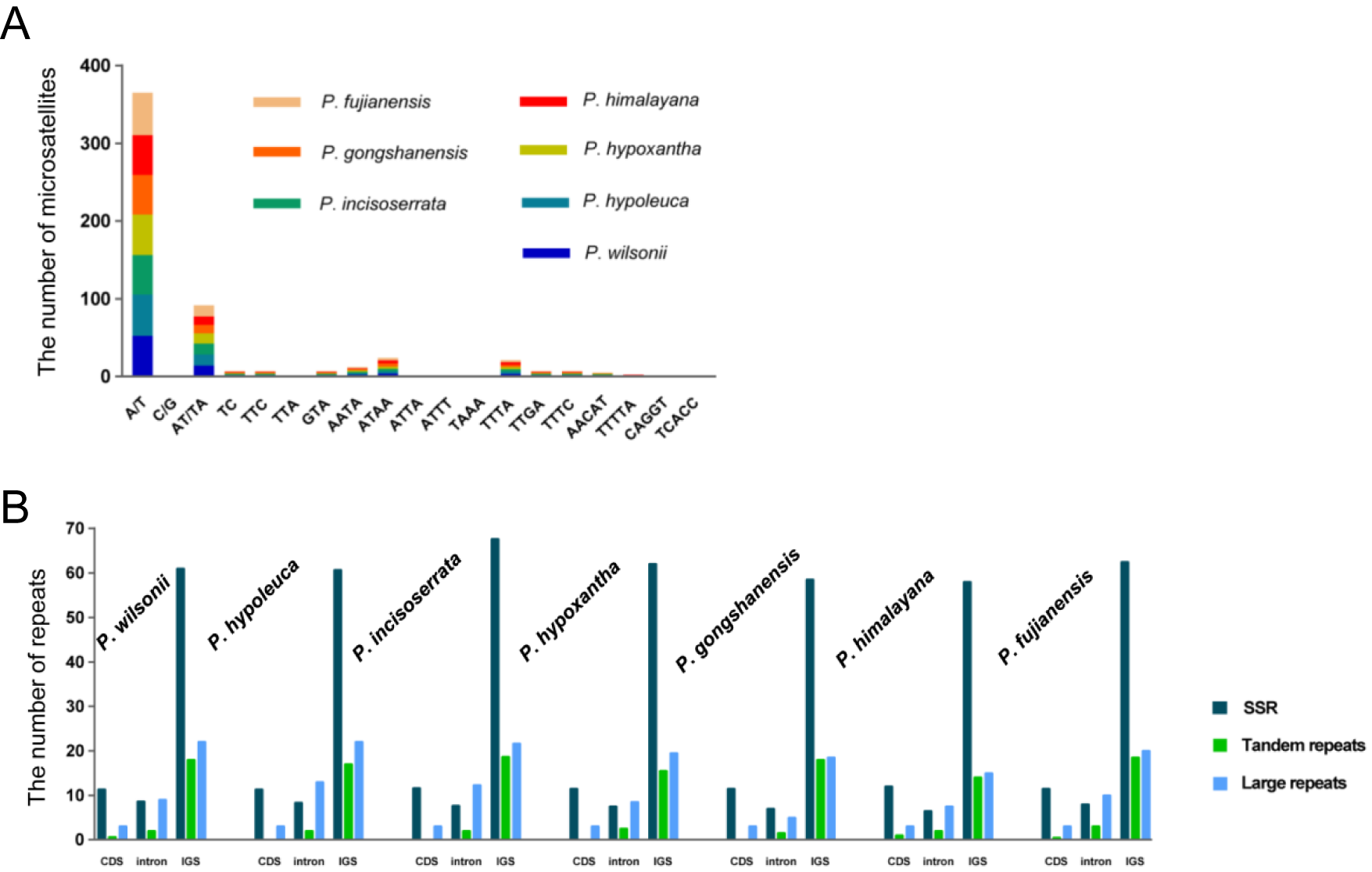

Supplement: Supplementary Figure 3 — Microsatellites number of different base compositions and distribution of all repeated sequences. (A) Frequency of microsatellites by base composition; (B) Number of all repeats by location. CDS, coding sequences; IGS, intergenic spacers. [file Image_3.TIF]

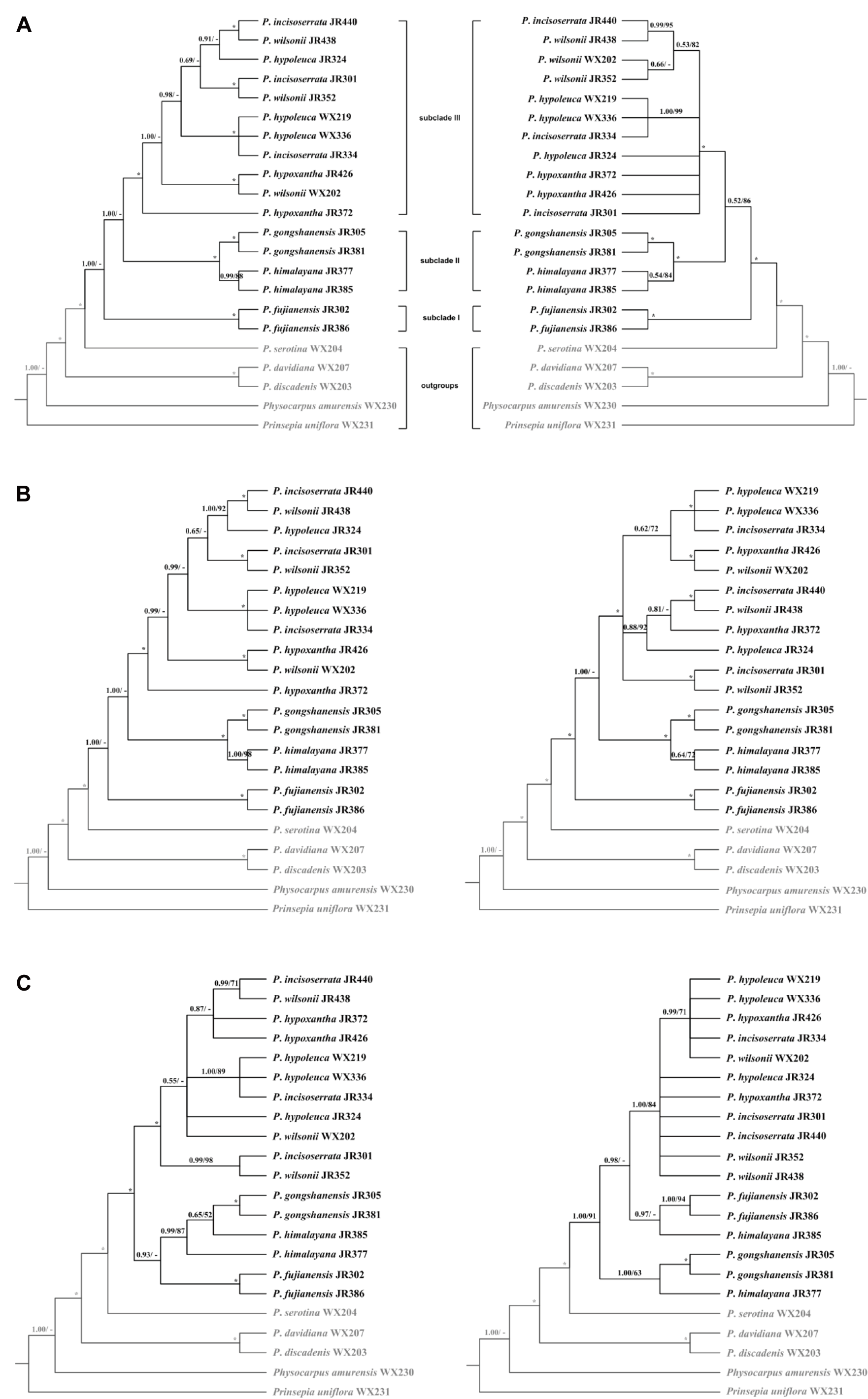

Supplement: Supplementary Figure 4 — Phylogenetic relationships of Maddenia inferred from Bayesian inference (BI) and maximum likelihood (ML) based on six datasets. (A) complete plastomes; (B) coding regions; (C) LSC region; (D) non-coding regions; (E) SSC region; (F) IR region. The support values above the branches show PP (posterior probability) / BS (bootstrap support), and asterisks indicate 1.00/100%. Dashes represent incongruences of BI and ML trees. [file Image_4.TIF]

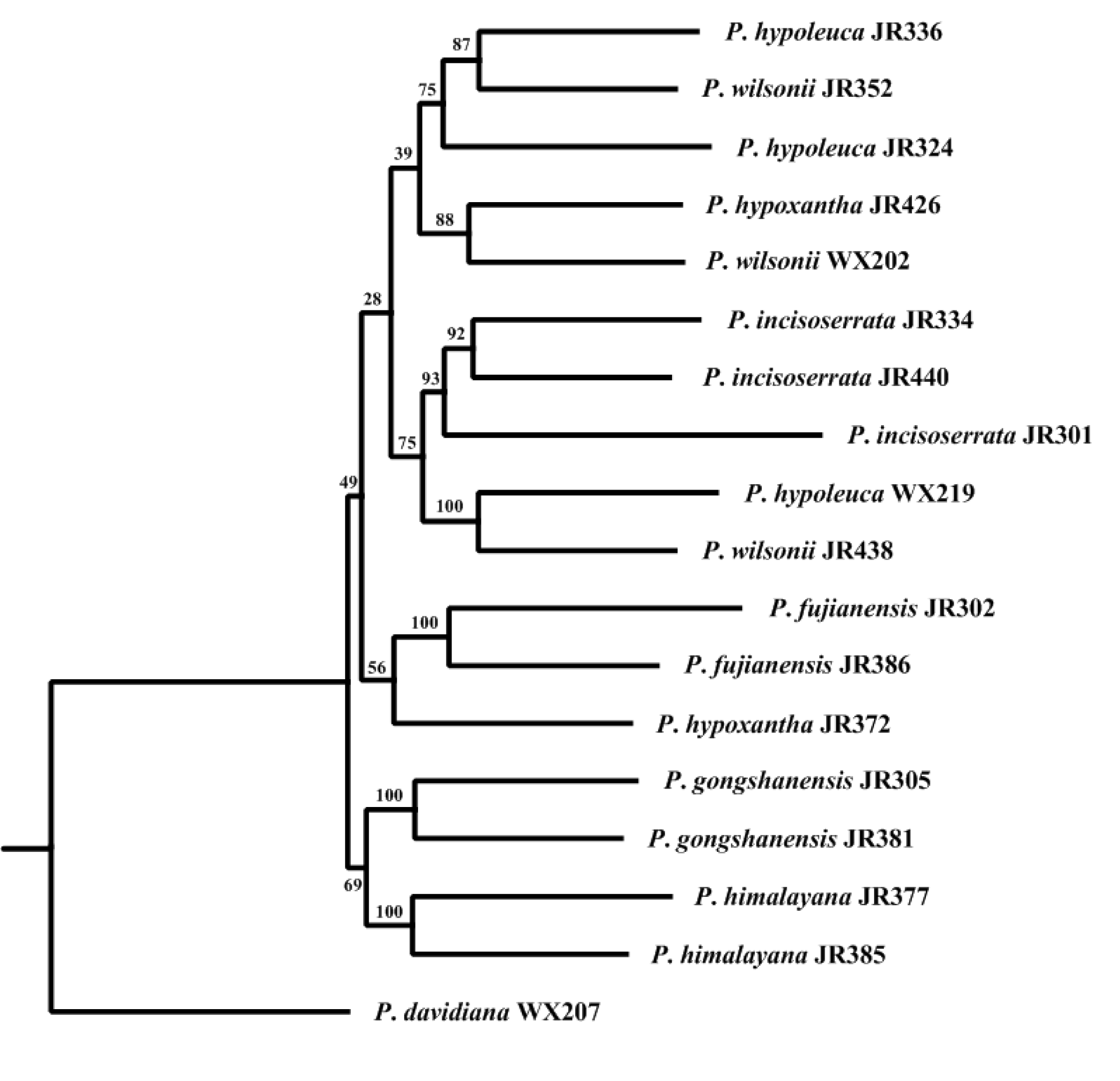

Supplement: Supplementary Figure 5 — Maximum likelihood inferred from 413 single-copy nuclear (SCN) genes of Maddenia group of Prunus. The number above branch indicated bootstrap support from the IQ-TREE. [file Image_5.TIF]

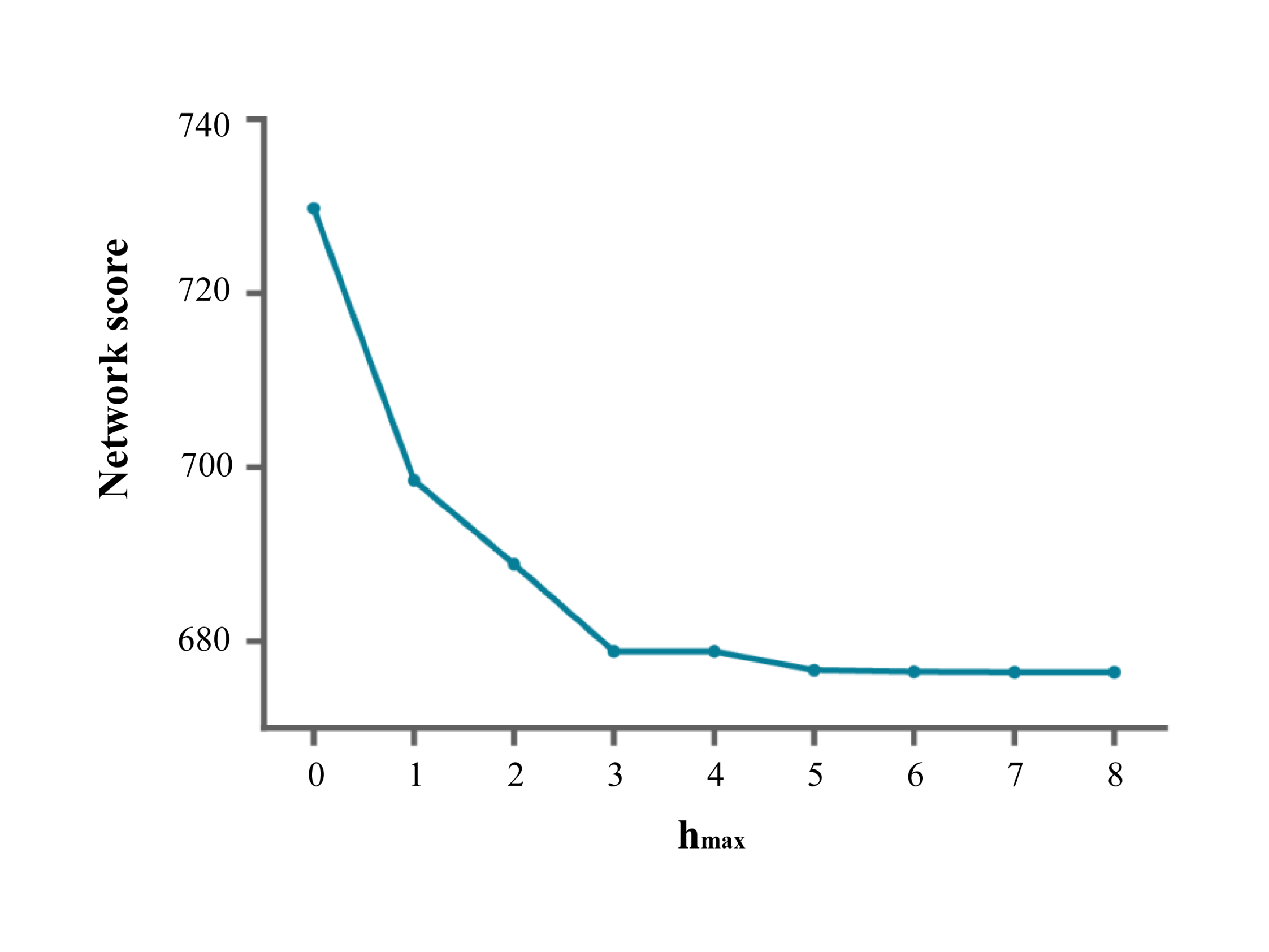

Supplement: Supplementary Figure 6 — The pseudolikelihood network score from the Species Networks applying Quartets (SNaQ) analysis for each of the maximum number of hybridizations allowed (hmax). [file Image_6.TIF]
