# Supplementary material for: On the Species Delimitation of the Maddenia Group of Prunus (Rosaceae): Evidence From Plastome and Nuclear Sequences and Morphology
Source: Front Plant Sci. 2021 Oct 11;12:743643. doi: 10.3389/fpls.2021.743643 (PMC8542774; doi:10.3389/fpls.2021.743643)
Supplement: Supplementary Table 1 — Genes present in the 17 complete Maddenia chloroplast genomes. [file Table_1.DOC]

| Gene group | Gene name | | | | |
| --- | --- | --- | --- | --- | --- |
| Ribosomal RNA genes | *rrn16* (2) | *rrn23* (2) | *rrn4.5* (2) | *rrn5* (2) |  |
| Transfer RNA genes | *trnI-CAU* (2) | *trnI-GAU* (2) * | *trnL-UAA* * | *trnL-CAA* (2) | *trnL-UAG* |
|  | *trnR-UCU* | *trnR-ACG* (2) | *trnA-UGC* (2) * | *trnW-CCA* | *trnM-CAU* |
|  | *trnV-UAC* * | *trnV-GAC* (2) | *trnF-GAA* | *trnT-UGU* | *trnT-GGU* |
|  | *trnP-UGG* | *trnfM-CAU* | *trnG-GCC* | *trnG-GCC* * | *trnS-GGA* |
|  | *trnS-UGA* | *trnS-GCU* | *trnD-GUC* | *trnC-GCA* | *trnN-GUU* (2) |
|  | *trnE-UUC* | *trnY-GUA* | *trnQ-UUG* | *trnK-UUU* * | *trnH-GUG* |
| Small Subunit of ribosome | *rps2* | *rps3* | *rps4* | *rps7* (2) | *rps8* |
|  | *rps11* | *rps12* (2) * | *rps14* | *rps15* | *rps16* |
|  | *rps18* | *rps19* |  |  |  |
| Large Subunit of ribosome | *rpl2*(2) * | *rpl14* | *rpl16* | *rpl20* | *rpl22* |
|  | *rpl23* (2) | *rpl32* | *rpl33* | *rpl36* |  |
| DNA-dependent RNA polymerase | *rpoA* | *rpoB* | *rpoC1* * | *rpoC2* |  |
| Translational initiation factor | *infA* |  |  |  |  |
| NADH dehygrogenase | *ndhA* * | *ndhB* (2) * | *ndhC* | *ndhD* | *ndhE* |
|  | *ndhF* | *ndhG* | *ndhH* | *ndhI* | *ndhJ* |
|  | *ndhK* |  |  |  |  |
| Subunits of photosystem I | *psaA* | *psaB* | *psaC* | *psaI* | *psaJ* |
|  | *ycf3* ** | *ycf4* |  |  |  |
| Subunits of photosystem II | *psbA* | *psbB* | *psbC* | *psbD* | *psbE* |
|  | *psbF* | *psbH* | *psbI* | *psbJ* | *psbK* |
|  | *psbL* | *psbM* | *psbN* | *psbT* | *psbZ* |
| Subunits of cytochrome | *petA* | *petB* | *petD* | *petG* | *petL* |
|  | *petN* |  |  |  |  |
| Subunits of ATP synthase | *atpA* | *atpB* | *atpE* | *atpF* * | *atpH* |
|  | *atpI* |  |  |  |  |
| Large subunit of Rubisco | *rbcL* |  |  |  |  |
| Maturase | *matk* |  |  |  |  |
| Membrane protein | *cemA* |  |  |  |  |
| Protease | *clpP* ** |  |  |  |  |
| Subunit of acetyl-CoA | *accD* |  |  |  |  |
| C-type cytochrome synthesis gene | *ccsA* |  |  |  |  |
| Hypothetical reading frames | *ycf1* | *ycf2* (2) |  |  |  |

**Table S1.** Genes present in the 17 complete *Maddenia* chloroplast genomes.

The number in braces mean genes with two copies, and single and double asterisks indicate genes with one and two introns, respectively.
